# Regional and age-specific global trends associated with infectious diarrhea in children under 14 years old caused by pathogenic microorganisms in 2021

**DOI:** 10.3389/fmed.2025.1676249

**Published:** 2025-10-20

**Authors:** Tuanjie Wang, Li Wang, Xinquan Sang, Yishuai Ren, Tingting Xu, Qian Huang, Aiju Xiao, Weihong Lu, Haibin Li, Shujun Li, Xiangtao Wu

**Affiliations:** ^1^Department of Pediatrics, First Affiliated Hospital of Xinxiang Medical University, Weihui, Henan, China; ^2^School of Public Health, Xinxiang Medical University, Xinxiang, Henan, China

**Keywords:** Global Burden of Disease, diarrhea, children, age trends, pathogens, risk factors

## Abstract

**Objective:**

This study aimed to systematically analyze the morbidity and mortality of infectious diarrhea in children under 14 years old caused by pathogenic microorganisms globally and their temporal trends.

**Methods:**

This study was a retrospective cross-sectional analysis using the database provided by the Global Burden of Disease 2021 (GBD 2021). The mortality, morbidity, and disability-adjusted life years (DALYs) of childhood diarrhea from 1990 to 2021 were stratified and analyzed using multivariate regression models and the calculation of estimated annual percentage change (EAPC).

**Results:**

In 2021, the total number of cases of diarrhea in children aged 0–14 years worldwide was 168.73 million. The number of cases was highest in low- and medium-SDI regions, and the incidence in South Asia was ranking first in the world. The highest incidence was in neonates, while the incidence in the 10–14 year old group has increased by 36.5% since 1990. The highest mortality rate was found in low SDI areas. In terms of risk factors, multivariate regression analysis showed that unsafe water sources were the most important risk factors for all age groups, and growth retardation in children and unsafe sanitation conditions also significantly increased the burden of diarrhea. Except for the 2–4 age group, the top three pathogens causing death and DALY in other age groups were *Rotavirus*, *Adenovirus*, and *Shigella*, with *Rotavirus* being the primary pathogen in all age groups.

**Conclusion:**

Although the global burden of diarrhea has decreased significantly, it is still high in low-income countries and low- and medium-SDI areas, and newborns are at high risk. Unsafe water sources are the main risk factor for diarrheal deaths in children of all ages.

## Introduction

The clinical symptoms of diarrhea include the passage of loose or watery stools, usually at least three bowel movements within 24 h, or a higher frequency than an individual’s normal bowel movement frequency. It remains one of the major threats to the health of children aged 0–14 years worldwide. Although the morbidity and mortality rates have declined, they remain severe (1–3). According to the Global Burden of Disease Study (GBD), diarrhea was still the fifth leading cause of death among children worldwide in 2016, especially among children under 5 years old (2, 4). The long-term harm of diarrhea to children’s health is not limited to death, but also includes negative impacts on nutrition, development and growth (5). From a geographical perspective, there are obvious differences in sanitary conditions, nutritional status and medical intervention measures in different regions, resulting in significant differences in the morbidity and mortality of diarrhea around the world (6, 7).

However, the downward trend in diarrhea is uneven, and the incidence and mortality of diarrhea among children in low-income and lower-middle-income countries remain high (7, 8). The Global Burden of Disease Study pointed out that the health losses caused by diarrhea in these countries are not only reflected in mortality, but also lead to a significant increase in DALYs (disability-adjusted life years) (8).

The triggering factors of diarrhea are mainly divided into two major categories. Infectious factors include infections by pathogens such as bacteria, viruses, and parasites, as well as other microorganisms. Non-infectious factors cover diet-related problems, intestinal function and diseases, the impacts of drugs and treatments, and systemic diseases and abnormal physiological states. Pathogenic studies have shown that diarrhea can be caused by a variety of infectious agents, with the most common pathogens including *Rotavirus*, *Cryptosporidium*, *Shigella*, *enterotoxigenic Escherichia coli (ETEC)*, *Adenovirus*, and *Norovirus*, among others. The prevalence of these pathogens varies by region and is closely associated with local sanitary conditions, nutritional status, and the availability of medical resources (9–13). Most of the existing literature focuses on diarrhea in children under 5 years old, but there is a relative lack of systematic, age-specific research on the incidence and mortality of diarrhea in children of all ages from 0 to 14 years old worldwide, which still leaves gaps or controversies in existing knowledge. In addition, there is a lack of in-depth discussion on the impact of certain specific pathogens such as *adenovirus* or enterotoxigenic *E. coli* on different age groups (7, 9). For example, *rotavirus* is still the main cause of hospitalization for diarrhea in many regions (10). Therefore, more detailed research is needed to clarify the changing trends in the burden of diarrhea among children of different age groups around the world and the high-risk areas/populations.

In addition, diarrhea not only affects children’s short-term health, but also has an adverse effect on their long-term growth and development. Studies have shown that diarrhea is closely related to growth retardation in children and may lead to a significant decrease in weight and height (6, 12). Therefore, further reducing the burden of diarrhea is crucial to the healthy growth of children worldwide (4, 7). Although the mortality rate from diarrhea has declined worldwide, it remains a major public health problem. So far, there has been no systematic study and report on the incidence and mortality of diarrhea in different age groups of children aged 0–14 years worldwide, and the high-risk causes and risk factors in different age groups.

The GBD database was selected for analysis because, on the one hand, it has a wide coverage of data, including high-quality data from multiple public health organizations, hospital records and large-scale epidemiological surveys around the world; on the other hand, GBD uses a series of rigorous methodologies, such as large-scale Bayesian hierarchical regression and comprehensive uncertainty assessment, which can accurately estimate the disease burden in different regions, ages and time dimensions. Through the analysis of the GBD database, we can have a more systematic understanding of the burden of diarrhea in children of different ages on a global scale.

Although there are discussions in the literature on developing targeted intervention strategies (5, 7), there is still a lack of detailed data to support intervention measures for high-burden countries and specific populations (such as newborns, low-income communities) or geographical regions (such as sub-Saharan Africa and South Asia). Based on the above background, this study will focus on the following questions: What is the spatiotemporal evolution of the incidence and mortality of diarrhea in children aged 0–14 years worldwide? What are the main pathogens and risk factors? Are there special heterogeneous burdens in different sociodemographic index (SDI) regions? By answering these questions, the study can provide a basis for the formulation of health policies in high-burden areas.

In summary, this article mainly focuses on digestive tract diseases in children caused by pathogenic microorganisms, and will follow the idea of “problem introduction - > limitations of existing research - > objectives and importance of this study.” Through the GBD 2021 data, the diarrhea burden of children aged 0–14 years will be deeply discussed, and corresponding health intervention strategy recommendations will be put forward. It is of great significance to further reduce global child diarrhea deaths, improve child nutrition and growth and development, and achieve broader child health goals.

## Research methods

### Study subjects

This study is based on data from the 2021 GBD study and aims to systematically analyze the incidence, mortality, disability-adjusted life years (DALYs), pathogens, and influencing factors of diarrhea in children aged 0–14 years of age of different genders in 204 countries and regions around the world from 1990 to 2021. The data of the GBD project mainly come from public health organizations, hospital records, epidemiological surveys, etc. of various countries, covering the incidence, prevalence, mortality, and health-related risk factors of various diseases. In GBD 2021, the world is divided into 7 GBD super regions and 21 GBD regions. For more details, see the relevant literature (13, 14). The data of the GBD study can be obtained from the Global Health Data Exchange (GHDx) platform^1^.

### Study groups

Age groups: newborns (0–28 days), 1–5 months, 6–11 months, 1–4 years, 5–9 years, and 10–14 years. SDI groups: According to SDI (sociodemographic index), 204 countries and regions in the world are divided into five groups: low SDI group (0 < SDI ≤ 0.570), low-medium SDI group (0.570 < SDI ≤ 0.670), medium SDI group (0.670 < SDI ≤ 0.812), high-medium SDI group (0.812 < SDI ≤ 0.858), and high SDI group (0.858 < SDI ≤ 1). The SDI value is a combination of per capita income, average education level, and fertility rate, and can better reflect the level of socioeconomic development (13). Inclusion criteria: We mainly included all available data on diarrhea in the GBD database and classified them according to the age group of 0–14 years. Exclusion criteria: If some countries or regions have no valid data or serious data missing during the period 1990–2021, statistical modeling cannot be carried out, they will be excluded.

### Data screening and processing

Before the formal analysis, the data was systematically cleaned and preprocessed to exclude duplicate or unreasonable values, and multiple interpolation was used to handle missing values. To ensure the robustness of the analysis results, residual and outlier detection was also performed on abnormal values. For time series data from different countries, regions and age groups, a stratified weighted method was used to summarize them to reduce the bias that may be caused by small-scale data.

## Analytical methods

### DALY and mortality estimates

The GBD database uses a Bayesian large-scale iterative hierarchical regression tool to generate internally consistent incidence and prevalence estimates. This study estimated diarrhea-related DALY and mortality based on this tool and analyzed them by multiple stratifications such as age, sex, and region. The results are expressed as 95% uncertainty intervals (UI).

### Analysis of major pathogens

The burden of multiple pathogens, including *norovirus*, *rotavirus*, *E. coli*, *Salmonella*, and *Shigella*, in different age groups was assessed, and the contribution of deaths and DALYs caused by each pathogen was calculated to identify the main intervention targets.

### Risk factor analysis

Conduct a systematic assessment of major risk factors such as unsafe drinking water, lack of sanitation facilities, malnutrition, low immunity, and poverty, and quantify the impact of these risk factors on the burden of diarrhea and the changing trends in different age groups and regions.

### Time trend analysis

Using historical data from 1990 to 2021, the estimated annual percentage change (EAPC) was used to evaluate the temporal trend of diarrhea burden. The mortality rate and its corresponding age standardized rate (ASR) were used to evaluate the trend of diarrheal diseases. The temporal trend of the age-standardized mortality rate of diarrheal diseases from 1990 to 2019 was quantified using the estimated annual percentage change (EAPC). The EAPC was calculated using a regression model based on the natural logarithm of the ratio: y = *α* + *β* x + *ε*.

The EAPC is calculated as 100 × (exp(β) − 1), where x is the calendar year and y is ln(ASR). It can be obtained using a linear regression model, and its 95% confidence interval (CI) can also be derived. If the predicted value of EAPC and its lower limit of 95% CI are both greater than 0, the ASR is considered to be on an upward trend. Conversely, if the predicted value of EAPC and its upper limit of 95% CI are both less than 0, it indicates that the ASR is on a downward trend. Otherwise, the ASR is considered to remain stable over time.

### APC model

This study also used the APC (age-period-cohort) model based on the Poisson distribution to conduct a more in-depth decomposition analysis of mortality to examine the individual and interactive effects of age, period, and birth cohort on the frequency of diarrhea (15); and tested the goodness of fit of the model through R^2^ value, AIC/BIC index and residual analysis, and conducted a sensitivity analysis of the model assumptions to ensure the robustness of EAPC and APC estimates.

## Result

### Changes in the incidence of diarrhea in children aged 0–14 years from 1990 to 2021

**Figure 1:**
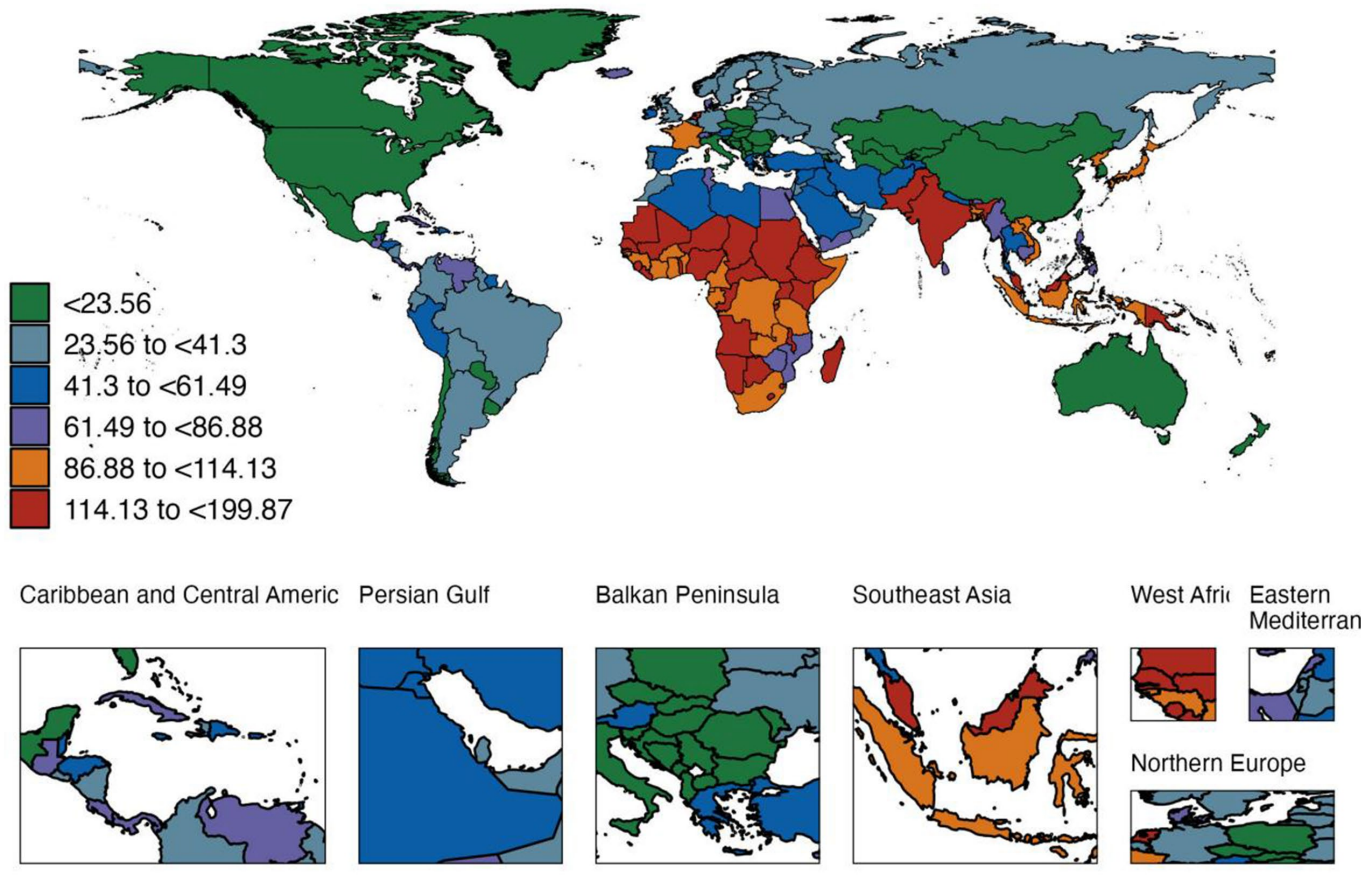
Diarrhea-related morbidity among children aged 0–14 years in 2021 (per 1,000,000,000).

In 2021, there were 168.73 million cases of diarrhea in children aged 0–14 years worldwide, with an incidence rate of 83,866.8 cases per 100,000 people (95% UI, [66,140.6–101,854.1]). The number of cases and incidence rates of diarrhea were highest in low- and medium-SDI regions (57.73 million), and after stratification by the World Bank, the number of cases was also highest in low- and middle-income countries (116.63 million), with an incidence rate of 115,156.0 cases per 100,000 people. After stratification by GBD super-region, South Asia ranked first in the world in both the number of cases and incidence rates (Supplementary Table 1 and Figure 1). Among 204 countries (or areas), the incidence of diarrhea in children aged 0–14 years ranged from 895.2 per 100,000 people (95% UI, [707.6–1136.7]) in the United States to 199,871.6 per 100,000 people (95% UI, [178,926.1–217,359.7]) in Madagascar in 2021. In terms of age groups, the highest incidence was in neonates (138,058.7 per 100,000 people), followed by children aged 1–5 months (117,600.4 per 100,000 people), and the lowest incidence was in children aged 2–4 years (47,673.5 per 100,000 people), and the incidence in the 10–14 year group increased again (106,298.1 per 100,000 people). Since 1990, the global incidence of diarrhea among children aged 0–14 has decreased by about 136%. However, the diarrhea rate among children aged 10–14 has increased by 36.5%, suggesting that there may be new dietary habits or lifestyle risks in adolescence. Supplementary Table 1 details the number of cases, incidence rates and EAPC changes in different age groups, SDI levels and World Bank classifications.

### Trends in deaths from diarrhea in children aged 0–14 years from 1990 to 2021

In 2019 (before the COVID-19 epidemic), 44,880 children aged 0–14 years died of diarrheal diseases (95%UI, [3.469–5.751]), with a mortality rate of 22.374 per 100,000 people (95%UI, [17.294–28.672]), a decrease of 474.8% since 1990. The largest decrease was in the neonatal age group, reaching 612.6%.

**Figure 2:**
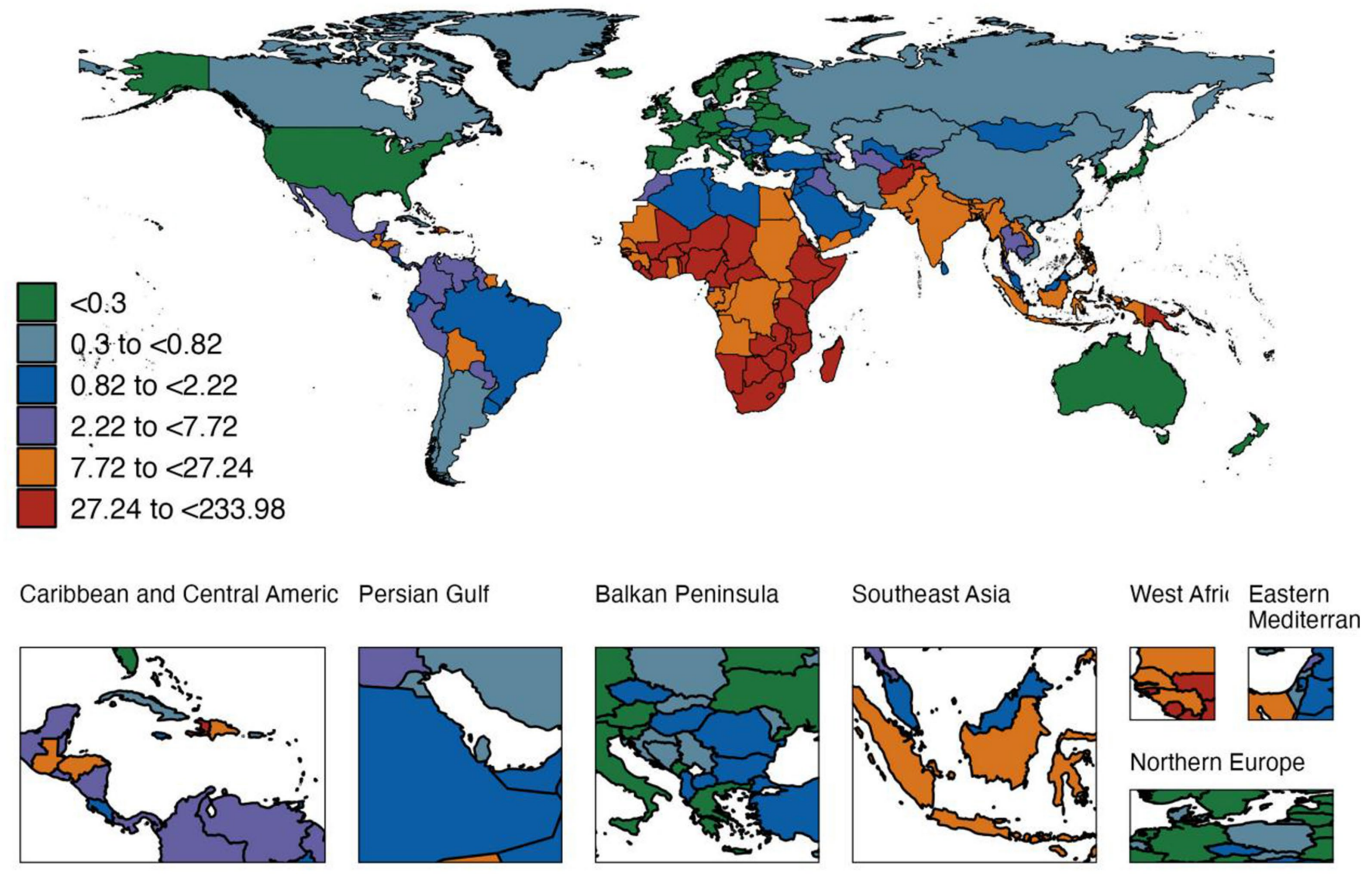
Global map of diarrhea mortality rates among children aged 0–14 years in 2021.

In 2021, diarrheal diseases caused 4,000 deaths (0.004 million) in children aged 0–14 years worldwide, with a mortality rate of 18.602 per 100,000 people (95% UI, [13.994–24.787]); the highest rate was in low SDI areas (53.3 times per 100,000 people), which was closely related to the continued existence of risk factors such as inadequate water safety and poor sanitation (Supplementary Table 2 and Figure 2). Among the 204 countries (or regions), the mortality rate ranged from 0.010 per 100,000 people in Andorra to 233.976 per 100,000 people in Chad. Newborns still had the highest mortality rate (251.1 per 100,000 people).

**Figure 3:**
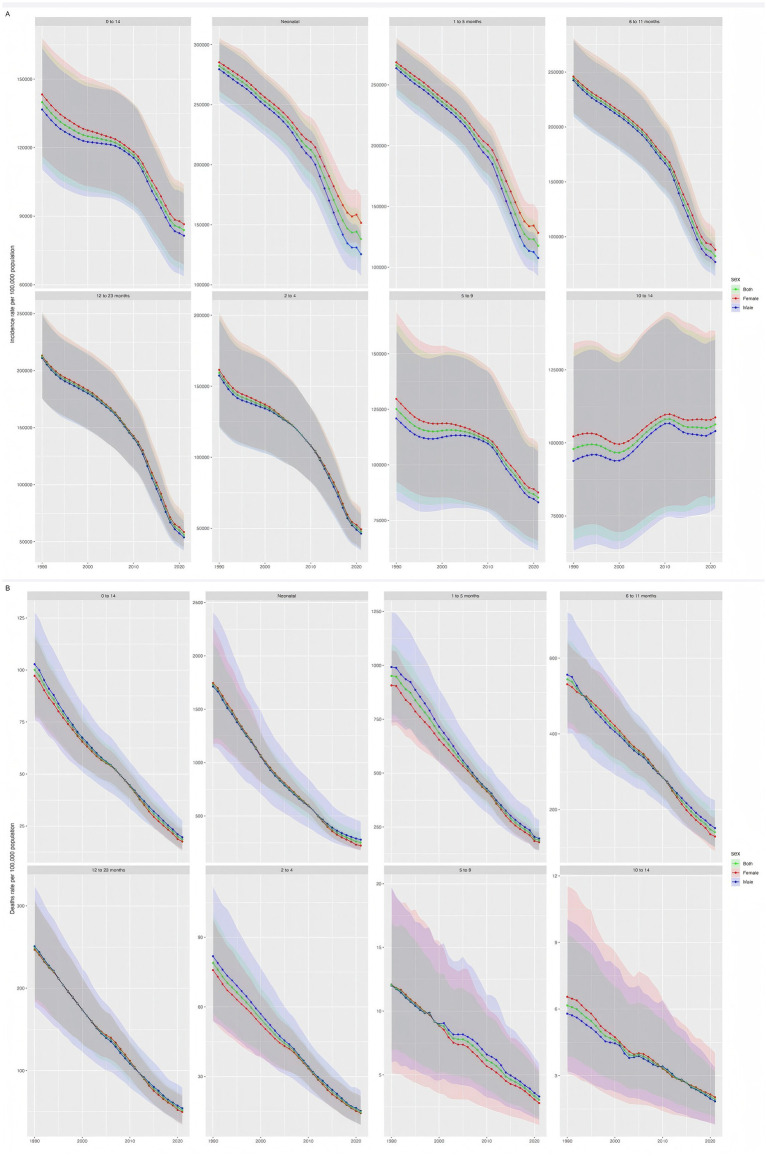
Global morbidity and mortality rates of diarrheal diseases among children aged 0–14 years, 1990–2021. Panel **(A)** shows the global incidence of diarrheal diseases among children aged 0–14 years old from 1990 to 2021; Panel **(B)** shows the global mortality rate of diarrheal diseases among children aged 0–14 years old from 1990 to 2021. The shaded area represents the 95% uncertainty interval.

Compared with 1990, the global diarrhea mortality rate among children aged 0–14 years has dropped significantly in all age groups, with the largest drop in newborns (85.5%), which may be related to the combined effects of probiotics application and improved neonatal intensive care. However, the decline in the 10–14 age group is relatively the smallest, suggesting that the prevention and intervention of diarrhea in adolescents should be further strengthened (Figure 3).

### Changes in the incidence and mortality of diarrheal diseases among children aged 0–14 years in different age groups from 1990 to 2021

Overall, between 1990 and 2021, the diarrhea mortality rate among children aged 0–14 years decreased by 81.4%, with females slightly higher than males; the incidence rate decreased by about 136%. Newborns and infants aged 1–5 months showed the highest morbidity and mortality burden, but the improvement was most obvious in low- and middle-income countries and low SDI areas (Figure 3).

### Analysis of risk factors for diarrhea in children of different ages from 1990 to 2021

**Figure 4:**
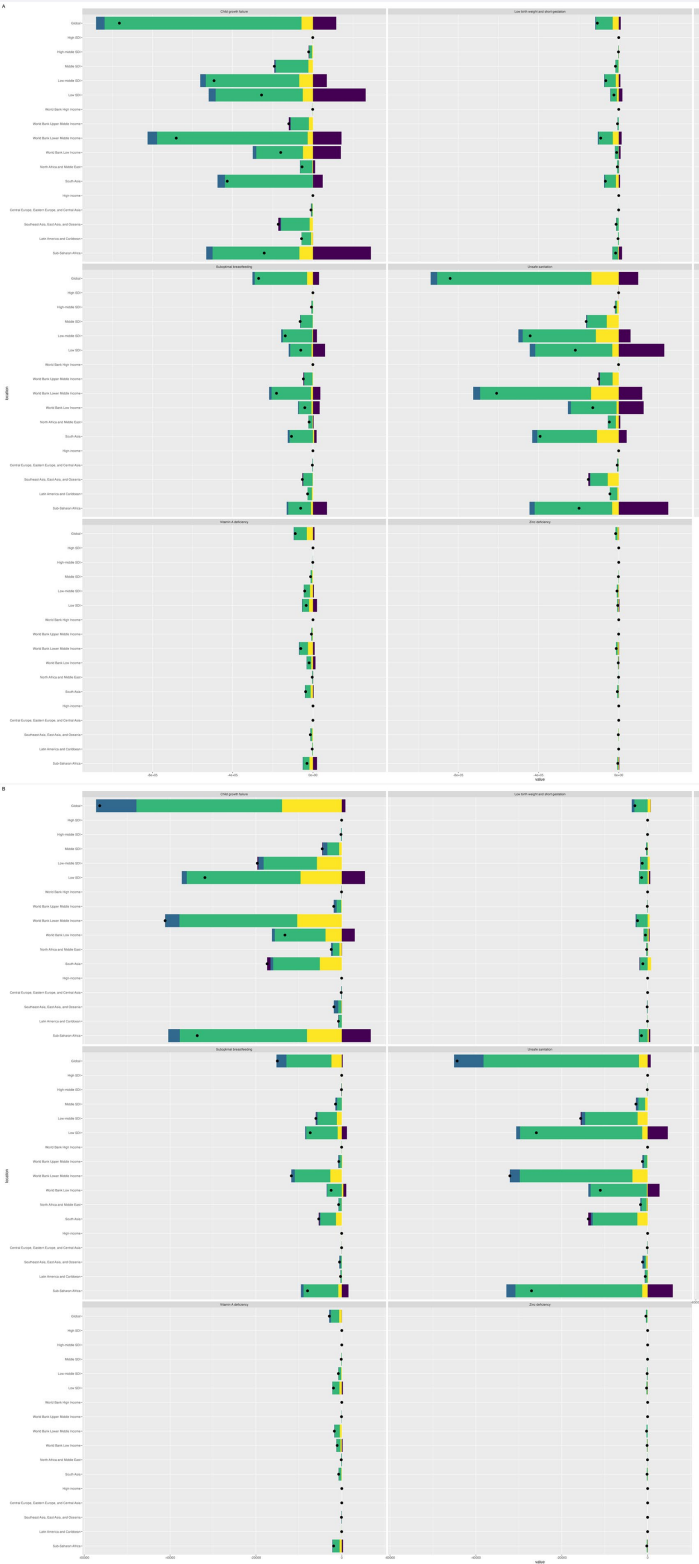
Analysis of risk factors for LRI in children aged 0–14 years. Panel **(A)** is, respectively, the analysis of risk factors for LRI in children aged 0–14 years from 1990 to 2019; Panel **(B)** is, respectively, the analysis of risk factors for LRI in children aged 0–14 years from 2019 to 2021.

In 2019, before the COVID-19 epidemic, the risk assessment attributed to diarrheal diseases showed that unsafe water sources, child growth retardation and unsafe sanitation were the top three factors. The number of DALYs caused by risk factors decreased by 463.3% compared with 1990, but it was still significant (Figure 4).

In 2021, the global burden of diarrhea in children aged 0–14 years is still mainly attributed to the above three risk factors; in terms of regions, in high SDI areas, child growth failure ranks first, while unsafe water sources are still the most prominent in low SDI areas. In addition, suboptimal breastfeeding has a greater impact on the newborn population, and Vitamin A deficiency also accounts for a certain proportion of children aged 2–4 years.

By age group, except for suboptimal breastfeeding in the neonatal stage which had a significant impact on diarrhea, unsafe water sources and unsafe sanitation conditions repeatedly appeared in the top three risks in other groups, once again emphasizing the importance of improving water, sanitation facilities and children’s nutritional status.

### Etiological distribution of DALYs and deaths from diarrheal diseases among children of different age groups worldwide in 2021

**Figure 5:**
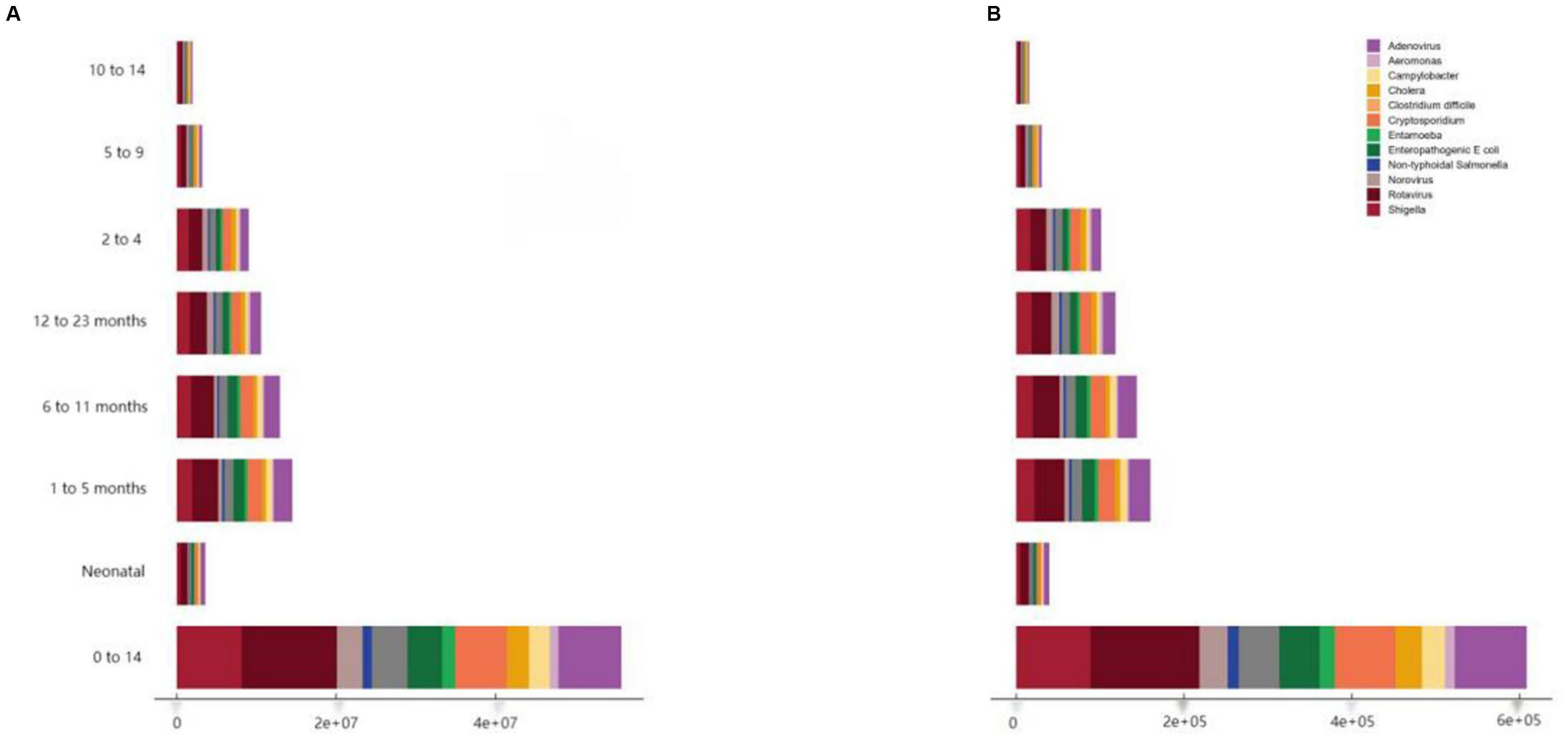
Etiological distribution of DALYs **(A)** and deaths **(B)** from diarrheal diseases in children worldwide by age group, 2021.

In 2021, *Rotavirus*, *Shigella*, and *Adenovirus* are the top three pathogens in most age groups. In terms of DALY, *rotavirus* is the highest (2.844 million), followed by *Shigella* (1.952 million) and *adenovirus* (2.023 million) (Figure 5). In high SDI areas, *rotavirus*, norovirus, and *Campylobacter* rank in the top three; in medium-high to medium-low SDI areas, *Adenovirus*’s contribution to diarrhea DALY has increased significantly; while in low SDI areas, *Rotavirus*, *Shigella*, and *Adenovirus* are still the main pathogens. In terms of age groups, *Rotavirus* and *Adenovirus* have the highest incidence and mortality rates among newborns, infants aged 1–5 months, and infants aged 6–11 months; *Rotavirus*, *Shigella*, and *Adenovirus* are the top three common pathogens in children aged 12–23 months, 2–4 years, 5–9 years, and 10–14 years. It is worth noting that among people aged 2–4 and 10–14, attention should also be paid to pathogens related to water and food contamination, such as *Vibrio cholerae* (Cholera) and enterotoxigenic *E. coli* (ETEC).

### Ranking of pathogens causing diarrhea-related deaths in children worldwide, 1990, 2019, 2020 and 2021

**Figure 6:**
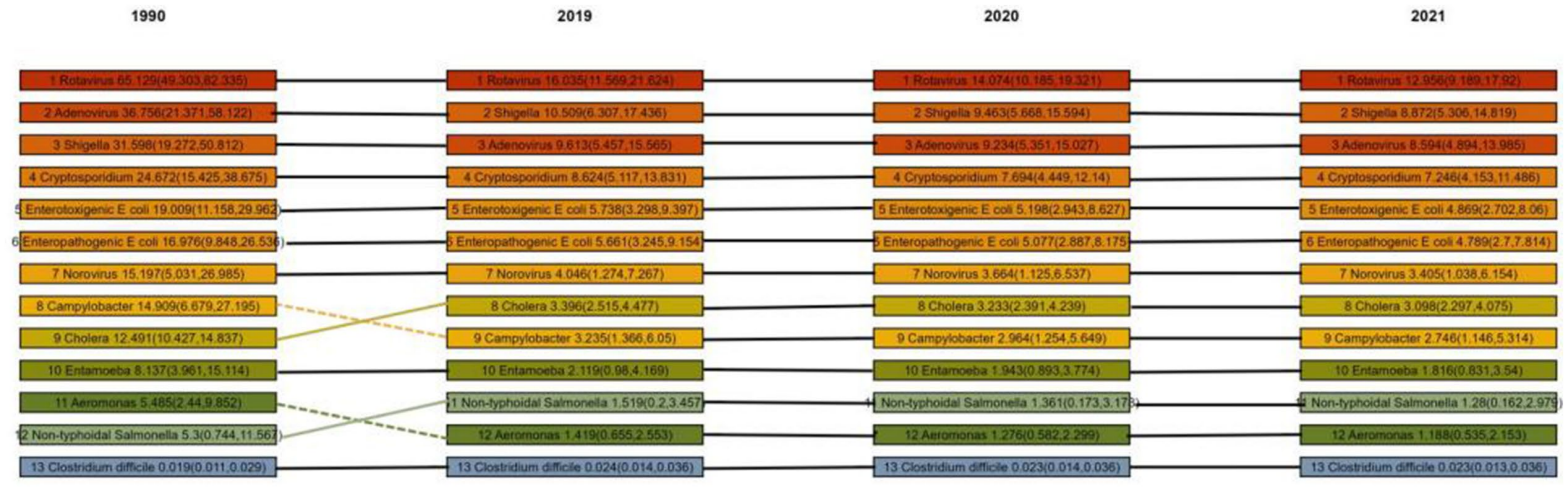
Shows the ranking of pathogens causing childhood diarrhea-related deaths worldwide in 1990, 2000, 2010 and 2021. Values are estimates of deaths (per 100,000 population) caused by each pathogen, with 95% uncertainty intervals in parentheses. Estimates are expressed to two significant figures.

In 2019, *Rotavirus*, *Shigella*, and *Adenovirus* were the top three causes of diarrheal death in children aged 0–14 years worldwide. Between 1990 and 2019, the global mortality rate caused by *Campylobacter* decreased the most (537.7%), followed by Cholera (534.1%), but *Clostridium difficile* increased significantly compared to 1990 (+53.1%). After 2021, the burden of diarrhea caused by major pathogens continued to decline, with the most significant decline being *Rotavirus* (about 19.2%) and the smallest being *C. difficile* (about 2.2%). Under the SDI quintile or World Bank level classification, *rotavirus* is the leading cause of death in almost all countries/regions, but the second and third causes may become *C. difficile*, *norovirus*, etc. in high SDI areas (Figures 5, 6). Therefore, differentiated intervention strategies should be adopted according to the etiological characteristics of different regions. Overall, the global non-COVID-19 diarrheal disease burden in children aged 0–14 years decreased significantly from 2019 to 2021. We estimated that the total incidence rate decreased by 2.0% (0.6–3.4), from 85844.241 (95% CI, [67922.822–103718.798]) to 83866.836 (66140.642–101854.133) per 100,000 population, and the DALYs rate decreased by 15.9% (10.9–20.6), from 2122.155 (95% CI, [1668.697–2670.056]) to 1784.278 (95% CI, [1361.382]) –The mortality rate decreased by 16.9% (11.3–22.0), from 22.374 cases per 100,000 people (95% CI, [17.294–28.672]) to 18.602 cases per 100,000 people (95% CI, [13.994–24.787]).

## Discussion

This study revealed that the morbidity and mortality of diarrhea in children aged 0–14 years have decreased significantly in the past few decades. However, the burden differences between different regions, genders and age groups are still significant, especially in low-income and low-medium SDI areas. This article further explores the important role of different age groups, geographical regions, pathogens and risk factors in the burden of childhood diarrhea, providing a more comprehensive perspective for understanding the current status and changing trends of the global burden of childhood diarrhea, and also provides data support for future intervention measures and resource allocation in high-burden areas.

In 2021, the global incidence of diarrhea in children aged 0–14 years decreased by 136%. The incidence and morbidity of diarrhea in low- and medium-SDI regions remained the highest, reaching 57.73 million cases. Although the burden of diarrheal diseases has decreased globally since 1990, the extent of the reduction has varied in different regions. Diarrhea-related mortality is highest in Asian and African countries (16). Before 2010, Asia was the main region for diarrheal deaths, but after 2011, the focus shifted to Africa (16). According to the 2021 GBD data, the incidence and morbidity of childhood diarrhea in South Asia were the highest, reaching 71.1 million cases, with an annual incidence of 140,235.1 cases per 100,000 people. In particular, in countries such as India and Pakistan, diarrhea is one of the main causes of child mortality (17, 18). Studies have shown that the incidence of diarrhea in rural and peri-urban populations in Cambodia is 281.5 per 1,000 people per year, and unemployment increases the risk of diarrhea by 63% (19). Fagbamigbe A’s study also showed that the overall prevalence of diarrhea in children under 5 years old in low- and middle-income countries was 14.4%, with the highest prevalence among boys, infants, low birth weight infants, the population in the lower fifth of family income, and those with primary education (20). When compared with other similar studies, it can be seen that: (1) This study found that the incidence of diarrhea in newborns was higher, which is different from some studies that emphasized that infants aged 6–11 months were most susceptible to the disease (21, 22). Infants in this age group are susceptible to infection because their immune systems are not fully developed. The incidence of children aged 12–23 months is still high, but lower than that of infants aged 6–11 months (23, 24). Children at this stage begin to be exposed to more solid food and environment, which increases the risk of infection. The incidence of children aged 24–59 months is significantly reduced, with an incidence of 1.9 times per child per year (24). At this time, the child’s immune system gradually matures and the resistance is enhanced. This study showed that the diarrhea rate of children aged 10–14 years has increased significantly. The increase in diarrhea rates among children aged 10–14 years has also been mentioned in other literature (25, 26). This suggests that changes in dietary behavior or poor weight control methods during adolescence may have an impact (27). In high SDI areas, *norovirus* and *Campylobacter* contribute more to diarrheal diseases, while in low SDI areas, *rotavirus* and *Shigella* are still the main pathogens (7). This is basically consistent with the distribution characteristics of major pathogens in different regions shown in the study by Kotloff et al. (10). The following biological and socioeconomic mechanisms behind the above findings can be inferred: the intestinal barrier and immune function of newborns are not yet sound, making them easily breached by *rotavirus* or *adenovirus*; the 10–14 year old group may be exposed to more out-of-school food or have unreasonable dieting/eating habits during adolescence, thereby increasing the incidence of diarrhea; and unsafe water sources and malnutrition in poor areas form a vicious cycle, further aggravating the burden of diarrhea. The above mechanism is closely related to the combined effects of improved sanitary conditions, vaccine promotion, and infant feeding methods.

In 2016, diarrhea was the eighth leading cause of death in all age groups worldwide and the fifth leading cause of death in children under 5 years of age (28). In 2021, the number of deaths from diarrhea in children aged 0–14 years worldwide was 0.004 million, with a mortality rate of 18.6 deaths per 100,000 people. Compared with 1990, the mortality rate has decreased by 474.8%. Despite the decline in mortality, diarrhea still accounts for 21% of deaths in children under 5 years of age (17). The mortality rate from diarrhea is highest in Africa, while it is lowest in Europe. A study pointed out that one in 10 deaths in children under 5 years of age is due to diarrhea, and the mortality rate has decreased the most in South Asia, Southeast Asia, and South America (7). This study showed that the mortality rate in the neonatal group decreased the most, which may be related to the use of probiotics and the improvement of nursing level. The study found that probiotic supplementation significantly reduced the mortality and morbidity of necrotizing enterocolitis in premature infants (29). Although low and lower-middle SDI countries had the highest burden in 2021, these countries also showed the greatest improvement in all-age mortality rates over time. The results of this study showed that the main risk factors for childhood diarrhea include unsafe water sources, poor child growth, and unsafe sanitation facilities. High incidence of diarrhea is closely related to lack of safe water sources and sanitation facilities, which is particularly evident in low-income and lower-middle-income countries (7, 19, 20). In low-income and middle-income countries, risk factors for diarrheal deaths include younger age (especially under 6 months), female gender, persistent diarrhea, and severe dehydration (30). Child growth failure is the leading risk factor for diarrhea in high SDI areas, while unsafe water sources are the leading risk factor in low SDI areas. Studies have shown that improving child nutrition can reduce diarrhea-related deaths and DALYs (31). High diarrhea prevalence and linear growth retardation in urban children in Dhaka, Bangladesh are associated with poor cognitive development outcomes (32). Unsafe drinking water significantly increases the risk of diarrhea in low-income and middle-income countries (33–35). Improving the safety of drinking water can significantly reduce the incidence of diarrhea (34). Poor economic conditions in poor areas limit the ability of families to access medical services and improve living conditions, thereby increasing the incidence of diarrhea (8). Improving sanitation facilities and promoting good hygiene habits can effectively reduce the incidence of diarrhea (33, 35). Climate change mainly refers to changes in temperature and precipitation (36), which will also affect the incidence of diarrhea, especially in areas with poor water sources and sanitation facilities (34). In South Asia and South America, the decline in diarrhea mortality has been particularly significant, which is closely related to the improvement of sanitation conditions in these regions (7). Community-led health interventions, such as the Community Total Health Program implemented in Ethiopia, have significantly reduced the incidence of diarrhea in children (37). Therefore, the World Health Organization has been working hard to promote improvements in water, sanitation and hygiene facilities (WaSH) to reduce the incidence of diarrhea (38). In addition, studies have shown that the incidence of diarrhea in children is higher in families with lower mothers’ education. Therefore, improving the education level of caregivers and improving family hygiene habits can also help reduce the risk of diarrhea (35).

In this study, in 2021, the main pathogens causing diarrhea in the world and in low SDI regions were *rotavirus*, *Shigella*, and *adenovirus*. In high SDI regions, the top three pathogens associated with the burden of DALYs from diarrheal diseases in children aged 0–14 years were *rotavirus*, *norovirus*, and *Campylobacter*. The pathogens of diarrheal deaths varied among different age groups, with *rotavirus* being the main pathogen in multiple age groups, except for children aged 2–4 years. The causes of death in children aged 2–4 years were affected by *rotavirus*, *adenovirus*, and *Shigella*, in order, as well as enterotoxigenic *E. coli*, *Aeromonas hydrophila*, and *V. cholerae*, which are usually associated with water contamination and poor food hygiene (5). For children aged 10–14 years, *rotavirus*, *Shigella*, and *V. cholerae* were the main pathogens causing diarrheal deaths, with the latter two being more common in areas with poor sanitation (39).

long been a major cause of diarrhea worldwide, especially in low-income and middle-income countries, where it is one of the leading causes of diarrhea hospitalization and death in children under 5 years of age (11). This study also shows that *rotavirus* is the leading cause of diarrhea in children of all ages. Other studies have shown that *Shigella* is an important cause of diarrhea in children in low-income countries (11, 40). *Adenovirus* is also an important cause of diarrhea in some regions, although its impact may vary from region to region (41). Studies have shown that *Rotavirus*, *Shigella*, *Norovirus*, and *Adenovirus* are the main causes of diarrhea in low-income and lower-middle-income countries (11, 12, 42). However, these studies did not conduct age-stratified studies. In South Asia, *Shigella* and *rotavirus* are the main causes of diarrhea in children, especially in children under 5 years of age, where the infection rate of these pathogens is high (43, 44). Thus, *rotavirus* and *Shigella* are the leading causes of antibiotic-treated diarrhea in children under 5 years of age in low- and middle-income countries, highlighting the need for vaccination to reduce antibiotic exposure (25).

Even in high SDI areas, *rotavirus* remains one of the main pathogens causing diarrhea in children, although the introduction of vaccines has significantly reduced its impact (42). *Norovirus* and *Campylobacter* are also important pathogens causing diarrhea in high SDI areas, the latter of which is often associated with food and water contamination (45).

In summary, *rotavirus*, *Shigella*, *norovirus*, and *adenovirus* are the main pathogens that cause diarrhea in children. Vaccines and treatment measures against these pathogens are key to reducing the burden of diarrhea (11). Vaccination against major pathogens, such as *rotavirus* vaccine, can significantly reduce the morbidity and mortality of diarrhea (7, 11). Childhood diarrhea not only has an impact on health, but also imposes a burden on family finances. The temporary reduction in disability-adjusted life years due to diarrheal disease varies across regions, sexes, and age groups, highlighting the need for tailored solutions based on local and demographic factors (46). Since 1990, the global incidence of diarrhea in children aged 0–14 years has declined significantly, a trend that is closely related to the progress of global health interventions, such as oral rehydration salts and vaccination.

This study may have the following shortcomings: data coverage is limited, and the data quality in some low-income and remote areas is low, which may not fully reflect the actual burden of diarrheal diseases, resulting in insufficient representation of the results; the dynamic changes of risk factors have not been analyzed in depth. Although the main risk factors have been identified, the dynamic trends and impacts of these factors over time and socioeconomic development have not been fully explored. Due to reliance on statistical models, the results in some regions and years may have estimation biases, especially when the quality of data input is poor. Finally, different diagnostic detection methods were used in the included studies, which may affect the analysis results (for example, molecular detection of Cryptosporidium is more sensitive and specific than microscopic detection methods), and may further limit the assessment of the diarrhea burden.

## Conclusion

Since 1990, the global incidence and mortality of diarrhea have declined significantly, but newborns and infants are still the main groups of morbidity and mortality. The incidence and mortality of diarrhea in low-income and low SDI areas are still high, indicating that these areas still face huge health challenges. There is a need to strengthen infrastructure construction, improve the safety of water and sanitation facilities, and improve children’s nutrition. *Rotavirus* remains the main pathogen of diarrhea in children of all ages worldwide, while *Shigella* and *Adenovirus* also occupy an important position in different regions and age groups. Therefore, vaccination and treatment measures against these pathogens should continue to be promoted, and research on other pathogens should be strengthened.

## Data Availability

The original contributions presented in the study are included in the article/Supplementary material, further inquiries can be directed to the corresponding authors.

## Footnotes

^1^ https://collab2021.healthdata.org/gbd-results/

## References

[1] Kosek M Bern C Guerrant RL. The global burden of diarrhoeal disease, as estimated from studies published between 1992 and 2000. Bull World Health Organ. (2003) 81:197–204. PMID: 12764516 PMC2572419

[2] GBD 2015 Eastern Mediterranean Region Diarrhea Collaborators. Burden of diarrhea in the eastern Mediterranean region, 1990-2015: findings from the global burden of disease 2015 study. Int J Public Health. (2018) 63:109–21. doi: 10.1007/s00038-017-1008-z, PMID: 28776239 PMC5973974

[3] GBD 2017 Diarrhoeal Disease Collaborators. Quantifying risks and interventions that have affected the burden of diarrhoea among children younger than 5 years: an analysis of the global burden of disease study 2017. Lancet Infect Dis. (2020) 20:37–59. doi: 10.1016/S1473-3099(19)30401-3, PMID: 31678029 PMC7340495

[4] GBD Diarrhoeal Diseases Collaborators. Estimates of global, regional, and national morbidity, mortality, and aetiologies of diarrhoeal diseases: a systematic analysis for the global burden of disease study 2015. Lancet Infect Dis. (2017) 17:909–48. doi: 10.1016/S1473-3099(17)30276-1, PMID: 28579426 PMC5589208

[5] Pajuelo MJ Noazin S Cabrera L Toledo A Velagic M Arias L. Epidemiology of enterotoxigenic *Escherichia coli* and impact on the growth of children in the first two years of life in Lima, Peru. Front Public Health. (2024) 12:1332319. doi: 10.3389/fpubh.2024.1332319, PMID: 38584932 PMC10995271

[6] Khalil IA Troeger C Rao PC Blacker BF Brown A Brewer TG. Morbidity, mortality, and long-term consequences associated with diarrhoea from Cryptosporidium infection in children younger than 5 years: a meta-analyses study. Lancet Glob Health. (2018) 6:e758–68. doi: 10.1016/S2214-109X(18)30283-3, PMID: 29903377 PMC6005120

[7] Local Burden of Disease Diarrhoea Collaborators. Mapping geographical inequalities in childhood diarrhoeal morbidity and mortality in low-income and middle-income countries, 2000-17: analysis for the global burden of disease study 2017. Lancet. (2020) 395:1779–801. doi: 10.1016/S0140-6736(20)30114-8, PMID: 32513411 PMC7314599

[8] Demissie GD Yeshaw Y Aleminew W. Diarrhea and associated factors among under five children in sub-Saharan Africa: evidence from demographic and health surveys of 34 sub-Saharan countries. PLoS One. (2021) 16:e0257522. doi: 10.1371/journal.pone.0257522, PMID: 34543347 PMC8452002

[9] Sadeghi H Aslanimehr M Nikkhahi F Safari R Vafaie M Gholamzadeh Khoei S. A systematic review and Meta-analysis of Aeromonas-associated Diarrhea among children in Asia. Surg Infect. (2024) 25:538–45. doi: 10.1089/sur.2024.090, PMID: 39129456

[10] Kotloff KL Nataro JP Blackwelder WC Nasrin D Farag TH Panchalingam S. Burden and aetiology of diarrhoeal disease in infants and young children in developing countries (the global enteric Multicenter study, GEMS): a prospective, case-control study. Lancet. (2013) 382:209–22. doi: 10.1016/S0140-6736(13)60844-2, PMID: 23680352

[11] Cohen AL Platts-Mills JA Nakamura T Operario DJ Antoni S Mwenda JM. Aetiology and incidence of diarrhoea requiring hospitalisation in children under 5 years of age in 28 low-income and middle-income countries: findings from the global Pediatric Diarrhea surveillance network. BMJ Glob Health. (2022) 7:e009548. doi: 10.1136/bmjgh-2022-009548, PMID: 36660904 PMC9445824

[12] Kotloff KL Nasrin D Blackwelder WC Wu Y Farag T Panchalingham S. The incidence, aetiology, and adverse clinical consequences of less severe diarrhoeal episodes among infants and children residing in low-income and middle-income countries: a 12-month case-control study as a follow-on to the global enteric Multicenter study (GEMS). Lancet Glob Health. (2019) 7:e568–84. doi: 10.1016/S2214-109X(19)30076-2, PMID: 31000128 PMC6484777

[13] Troeger C Colombara DV Rao PC Khalil IA Brown A Brewer TG. Global disability-adjusted life-year estimates of long-term health burden and undernutrition attributable to diarrhoeal diseases in children younger than 5 years. Lancet Glob Health. (2018) 6:e255–69. doi: 10.1016/S2214-109X(18)30045-7, PMID: 29433665 PMC5861379

[14] Jin W Huang K Ding Z Zhang M Li C Yuan Z. Global, regional, and national burden of esophageal cancer: a systematic analysis of the global burden of disease study 2021. Biomark Res. (2025) 13:3. doi: 10.1186/s40364-024-00718-2, PMID: 39762900 PMC11702276

[15] Bender R Sirota S Swetschinski L Dominguez R-M Novotney A Wool E. Global, regional, and national incidence and mortality burden of non-COVID-19 lower respiratory infections and aetiologies, 1990-2021: a systematic analysis from the global burden of disease study 2021. Lancet Infect Dis. (2024) 24:974–1002. doi: 10.1016/S1473-3099(24)00176-2, PMID: 38636536 PMC11339187

[16] Rosenberg PS Anderson WF. Age-period-cohort models in cancer surveillance research: ready for prime time? Cancer Epidemiol Biomarkers Prev. (2011) 20:1263–8. doi: 10.1158/1055-9965.EPI-11-0421, PMID: 21610223 PMC3132831

[17] Almasi A Zangeneh A Ziapour A Saeidi S Teimouri R Ahmadi T. Investigating global spatial patterns of Diarrhea-related mortality in children under five. Front Public Health. (2022) 10:861629. doi: 10.3389/fpubh.2022.861629, PMID: 35910920 PMC9334699

[18] Kotloff KL Blackwelder WC Nasrin D Nataro JP Farag TH van Eijk A. The global enteric Multicenter study (GEMS) of diarrheal disease in infants and young children in developing countries: epidemiologic and clinical methods of the case/control study. Clin Infect Dis. (2012) 55:S232–45. doi: 10.1093/cid/cis753, PMID: 23169936 PMC3502307

[19] Kelly GC Rachmat A Hontz RD Sklar MJ Tran LK Supaprom C. Etiology and risk factors for diarrheal disease amongst rural and peri-urban populations in Cambodia, 2012–2018. PLoS One. (2023) 18:e0283871. doi: 10.1371/journal.pone.0283871, PMID: 37000848 PMC10065300

[20] Fagbamigbe AF Uthman AO Ibisomi L. Hierarchical disentanglement of contextual from compositional risk factors of diarrhoea among under-five children in low- and middle-income countries. Sci Rep. (2021) 11:8564. doi: 10.1038/s41598-021-87889-2, PMID: 33879839 PMC8058334

[21] Fischer Walker CL Perin J Aryee MJ Boschi-Pinto C Black RE. Diarrhea incidence in low- and middle-income countries in 1990 and 2010: a systematic review. BMC Public Health. (2012) 12:220. doi: 10.1186/1471-2458-12-220, PMID: 22436130 PMC3323412

[22] Salência-Ferrão J Chissaque A Manhique-Coutinho L Kenga AN Cassocera M de Deus N. Inappropriate use of antibiotics in the management of diarrhoea in children under five years admitted with acute diarrhoea in four provinces of Mozambique 2014-2019. BMC Infect Dis. (2025) 25:209. doi: 10.1186/s12879-025-10597-z, PMID: 39939844 PMC11823034

[23] Susilowati E Astuti Y Mulyasih R. Scoping review: diarrhea in toddlers and causing factors. PKM-P. (2023) 7:130. doi: 10.32832/jurma.v7i1.1708

[24] Lewnard JA Rogawski McQuade ET Platts-Mills JA Kotloff KL Laxminarayan R. Incidence and etiology of clinically-attended, antibiotic-treated diarrhea among children under five years of age in low- and middle-income countries: evidence from the global enteric Multicenter study. PLoS Negl Trop Dis. (2020) 14:e0008520. doi: 10.1371/journal.pntd.0008520, PMID: 32776938 PMC7444547

[25] Wiklund CA Kuja-Halkola R Thornton LM. Prolonged constipation and diarrhea in childhood and disordered eating in adolescence. J Psychosom Res. (2019) 126:109797. doi: 10.1016/j.jpsychores.2019.109797, PMID: 31536865

[26] de Souza ALG de Almeida AA Noll PRES Noll M. Unhealthy life habits associated with self-induced vomiting and laxative misuse in Brazilian adolescents. Sci Rep. (2021) 11:2482. doi: 10.1038/s41598-021-81942-w, PMID: 33510267 PMC7843628

[27] Yang K Kwon S Burton-Murray H Kuo B Chan AT Field AE. Maladaptive weight control and eating behaviours in female adolescents/young adults are associated with increased risk of irritable bowel syndrome in adulthood: results from the growing up today study (GUTS). Aliment Pharmacol Ther. (2024) 60:934–9. doi: 10.1111/apt.18197, PMID: 39102895 PMC11524775

[28] GBD 2016 Diarrhoeal Disease Collaborators. Estimates of the global, regional, and national morbidity, mortality, and aetiologies of diarrhoea in 195 countries: a systematic analysis for the global burden of disease study 2016. Lancet Infect Dis. (2018) 18:1211–28. doi: 10.1016/S1473-3099(18)30362-1, PMID: 30243583 PMC6202444

[29] Sharif S Meader N Oddie SJ Rojas-Reyes MX McGuire W. Probiotics to prevent necrotising enterocolitis in very preterm or very low birth weight infants. Cochrane Database Syst Rev. (2023) 7:CD005496. doi: 10.1002/14651858.CD005496.pub6 37493095 PMC10370900

[30] Hartman RM Cohen AL Antoni S Mwenda J Weldegebriel G Biey J. Risk factors for mortality among children younger than age 5 years with severe Diarrhea in low- and middle-income countries: findings from the World Health Organization-coordinated global rotavirus and Pediatric Diarrhea surveillance networks. Clin Infect Dis. (2023) 76:e1047–53. doi: 10.1093/cid/ciac561, PMID: 35797157 PMC9907489

[31] Behera DK Mishra S. The burden of diarrhea, etiologies, and risk factors in India from 1990 to 2019: evidence from the global burden of disease study. BMC Public Health. (2022) 22:92. doi: 10.1186/s12889-022-12515-3, PMID: 35027031 PMC8759196

[32] George CM Perin J Parvin T Bhuyian MSI Thomas ED Monira S. Diarrhea prevalence and child growth faltering are associated with subsequent adverse child developmental outcomes in Bangladesh (CHoBI7 program). Am J Trop Med Hyg. (2021) 106:233–8. doi: 10.4269/ajtmh.21-0767, PMID: 34724631 PMC8733517

[33] Geremew G Cumming O Haddis A Freeman MC Ambelu A. Rainfall and temperature influences on childhood Diarrhea and the effect modification role of water and sanitation conditions: A systematic review and Meta-analysis. Int J Environ Res Public Health. (2024) 21:823. doi: 10.3390/ijerph21070823, PMID: 39063400 PMC11276699

[34] Berendes DM Fagerli K Kim S Nasrin D Powell H Kasumba IN. Survey-based assessment of water, sanitation, and animal-associated risk factors for moderate-to-severe diarrhea in the vaccine impact on diarrhea in Africa (VIDA) study: the Gambia, Mali, and Kenya, 2015-2018. Clin Infect Dis. (2023) 76:S132–9. doi: 10.1093/cid/ciac911, PMID: 37074438 PMC10116493

[35] Mebrahtom S Worku A Gage D. The risk of water, sanitation and hygiene on diarrhea-related infant mortality in eastern Ethiopia: a population-based nested case-control. BMC Public Health. (2022) 22:343. doi: 10.1186/s12889-022-12735-7, PMID: 35177054 PMC8855567

[36] Colston JM Faruque ASG Hossain MJ Saha D Kanungo S Mandomando I. Associations between household-level exposures and all-cause Diarrhea and pathogen-specific enteric infections in children enrolled in five sentinel surveillance studies. Int J Environ Res Public Health. (2020) 17:8078. doi: 10.3390/ijerph17218078, PMID: 33147841 PMC7663028

[37] Dhimal M Bhandari D Karki KB Shrestha SL Khanal M Shrestha RRP. Effects of climatic factors on diarrheal diseases among children below 5 years of age at national and subnational levels in Nepal: an ecological study. Int J Environ Res Public Health. (2022) 19:6138. doi: 10.3390/ijerph19106138, PMID: 35627674 PMC9140521

[38] Cha S Jung S Bizuneh DB Abera T Doh YA Seong J. Effect of a community-led Total sanitation intervention on the incidence and prevalence of Diarrhea in children in rural Ethiopia: A cluster-randomized controlled trial. Am J Trop Med Hyg. (2021) 105:532–43. doi: 10.4269/ajtmh.20-0014, PMID: 34125700 PMC8437198

[39] Ashenafi G Tilahun D Aliyo A Sisay B. Magnitude of enteric pathogens associated with diarrhea and antibiotic resistance of enteric bacterial pathogens isolated among children under 5 years of age in bule Hora town, west Guji, Ethiopia. Front Public Health. (2024) 12:1398264. doi: 10.3389/fpubh.2024.1398264, PMID: 39435410 PMC11491406

[40] Tauheed I Ahmed T Akter A Firoj MG Ahmmed F Rahman SIA. A snap-shot of a diarrheal epidemic in Dhaka due to enterotoxigenic Escherichia coli and *Vibrio cholerae* O1 in 2022. Front Public Health. (2023) 11:1132927. doi: 10.3389/fpubh.2023.1132927, PMID: 37124777 PMC10140589

[41] Belay A Ashagrie M Seyoum B Alemu M Tsegaye A. Prevalence of enteric pathogens, intestinal parasites and resistance profile of bacterial isolates among HIV infected and non-infected diarrheic patients in Dessie town, Northeast Ethiopia. PLoS One. (2020) 15:e0243479. doi: 10.1371/journal.pone.0243479, PMID: 33320909 PMC7737993

[42] Nhampossa T Mandomando I Acacio S Quintó L Vubil D Ruiz J. Diarrheal disease in rural Mozambique: burden, risk factors and Etiology of diarrheal disease among children aged 0-59 months seeking Care at Health Facilities. PLoS One. (2015) 10:e0119824. doi: 10.1371/journal.pone.0119824, PMID: 25973880 PMC4431848

[43] Kawai K O’Brien MA Goveia MG Mast TC El Khoury AC. Burden of rotavirus gastroenteritis and distribution of rotavirus strains in Asia: a systematic review. Vaccine. (2012) 30:1244–54. doi: 10.1016/j.vaccine.2011.12.092, PMID: 22212128

[44] Muzembo BA Kitahara K Mitra D Ohno A Khatiwada J Dutta S. Burden of *Shigella* in South Asia: a systematic review and meta-analysis. J Travel Med. (2023) 30:taac132. doi: 10.1093/jtm/taac132, PMID: 36331282

[45] Platts-Mills JA Babji S Bodhidatta L Gratz J Haque R Havt A. Pathogen-specific burdens of community diarrhoea in developing countries: a multisite birth cohort study (MAL-ED). Lancet Glob Health. (2015) 3:e564–75. doi: 10.1016/S2214-109X(15)00151-5, PMID: 26202075 PMC7328884

[46] Karambizi NU McMahan CS Blue CN Temesvari LA. Global estimated disability-adjusted life-years (DALYs) of diarrheal diseases: a systematic analysis of data from 28 years of the global burden of disease study. PLoS One. (2021) 16:e0259077. doi: 10.1371/journal.pone.0259077, PMID: 34705878 PMC8550424

