# Supplementary material for: Regional and age-specific global trends associated with infectious diarrhea in children under 14 years old caused by pathogenic microorganisms in 2021
Source: Front Med (Lausanne). 2025 Oct 20;12:1676249. doi: 10.3389/fmed.2025.1676249 (PMC12580197; doi:10.3389/fmed.2025.1676249)
Supplement: Supplementary file 1 [file Table_1.docx]

Supplementary Table 1 Number of cases, incidence and annual percentage change of diarrheal diseases in children aged 0-14 years from 1990 to 2021

| Locat​  ion | 1990 | | 2019 | | 2020 | | 2021 | | Incidence rate change, % t | |
| --- | --- | --- | --- | --- | --- | --- | --- | --- | --- | --- |
|  | Incidence  counts | Incidence  rate per  100 000  population | Incidence  counts | Incidence  rate per  100 000  population | Incidence  counts | Incidence  rate per  100 000  population | Incidence  counts | Incidence  rate per  100 000  population | 1990-2019 | 2019-2021 |
| Global | | | | | | | | | | |
| 0 to 14 | 1739616.352(1373131.016 to 2048270.642) | 100.027(78.954 to 117.774) | 448812.517(346906.545 to 575140.621) | 22.374(17.294 to 28.672) | 400655.318(305575.673 to 525359.77) | 19.921(15.194 to 26.121) | 374245.623(281541.312 to 498683.54) | 18.602(13.994 to 24.787) | -4.704  (-4.926 to -4.482) | -0.023 (-0.037 to -0.009) |
| Neonatal | 173540.288(121913.402 to 213654.379) | 1728.563(1214.329 to 2128.123) | 28513.553(21872.155 to 38532.437) | 281.41(215.864 to 380.29) | 25796.823(19479.199 to 35511.495) | 260.843(196.963 to 359.073) | 24474.451(18314.396 to 34191.137) | 251.143(187.932 to 350.851) | -5.911  (-6.096 to -5.726) | -0.038(-0.051 to -0.025) |
| 1 to 5 months | 519111.891(420211.626 to 598302.318) | 950.829(769.679 to 1095.878) | 125358.67(99381.657 to 159257.145) | 223.924(177.522 to 284.476) | 106537.843(83491.517 to 135449.079) | 194.858(152.706 to 247.736) | 101180.492(78489.364 to 129893.636) | 188.218(146.008 to 241.631) | -5.021  (-5.256 to -4.785) | -0.046(-0.058 to -0.034) |
| 6 to 11 months | 343365.205(270491.54 to 410049.272) | 544.054(428.587 to 649.713) | 106894.081(79842.013 to 143958.109) | 161.884(120.915 to 218.015) | 95203.771(69664.723 to 131485.142) | 147.501(107.933 to 203.712) | 88678.832(63716.955 to 124323.263) | 140.332(100.83 to 196.738) | -3.836  (-4.027 to -3.646) | -0.072(-0.083 to -0.06) |
| 12 to 23 months | 310123.354(233713.307 to 381694.218) | 248.859(187.544 to 306.291) | 79985.945(58975.852 to 105260.729) | 59.555(43.911 to 78.374) | 72222.388(52188.922 to 96597.83) | 54.948(39.706 to 73.493) | 66885.999(47130.06 to 91541.737) | 52.088(36.703 to 71.288) | -4.691  (-4.884 to -4.497) | -0.111(-0.124 to -0.096) |
| 2 to 4 | 290173.63(210797.349 to 368655.352) | 78.943(57.349 to 100.295) | 69033.727(46757.325 to 97738.94) | 16.769(11.358 to 23.742) | 64670.734(42280.124 to 92970.861) | 15.802(10.331 to 22.718) | 59209.45(37892.313 to 86988.189) | 14.69(9.401 to 21.582) | -5.066  (-5.358 to -4.773) | -0.104(-0.125 to -0.085) |
| 5 to 9 | 70288.54(40219.911 to 99719.459) | 12.045(6.893 to 17.089) | 24787.54(14177.626 to 41362.336) | 3.67(2.099 to 6.125) | 22647.194(12583.74 to 38220.064) | 3.326(1.848 to 5.613) | 20943.363(11638.436 to 34889.208) | 3.048(1.694 to 5.078) | -3.559  (-3.827 to -3.29) | -0.026(-0.041 to -0.011) |
| 10 to 14 | 33013.443(20546.441 to 50102.056) | 6.163(3.836 to 9.353) | 14239.001(8401.941 to 23784.09) | 2.182(1.288 to 3.645) | 13576.566(8080.453 to 22446.815) | 2.055(1.223 to 3.398) | 12873.035(7498.045 to 21217.572) | 1.931(1.125 to 3.183) | -3.221  (-3.394 to -3.048) | 0.012(-0.003 to 0.029) |
| High SDI | | | | | | | | | | |
| 0 to 14 | 2207.306(1723.336 to 2935.859) | 1.188(0.927 to 1.58) | 571.461(518.62 to 632.697) | 0.328(0.298 to 0.363) | 504.365(448.591 to 563.29) | 0.291(0.259 to 0.325) | 474.56(410.766 to 539.035) | 0.275(0.238 to 0.312) | -4.243  (-4.5 to -3.985) | -0.043(-0.062 to -0.025) |
| Neonatal | 241.128(179.448 to 351.74) | 25.365(18.877 to 37.001) | 93.609(85.868 to 104.038) | 11.605(10.645 to 12.898) | 84.683(75.571 to 95.81) | 10.725(9.571 to 12.134) | 79.664(68.988 to 91.49) | 10.163(8.801 to 11.672) | -2.647  (-2.876 to -2.416) | -0.016(-0.044 to 0.012) |
| 1 to 5 months | 771.294(593.263 to 1073.841) | 14.704(11.31 to 20.472) | 199.003(182.195 to 223.105) | 4.429(4.055 to 4.965) | 183.899(162.477 to 206.609) | 4.186(3.698 to 4.703) | 172.1(146.314 to 198.16) | 3.959(3.366 to 4.558) | -4.641  (-4.827 to -4.455) | -0.02(-0.045 to 0.004) |
| 6 to 11 months | 539.841(355.533 to 810.142) | 8.789(5.788 to 13.19) | 89.694(76.955 to 106.078) | 1.678(1.44 to 1.985) | 71.047(58.693 to 85.675) | 1.362(1.126 to 1.643) | 65.897(53.284 to 79.988) | 1.285(1.039 to 1.56) | -5.294  (-5.694 to -4.893) | -0.027(-0.056 to -0.002) |
| 12 to 23 months | 385.967(276.538 to 567.933) | 3.128(2.241 to 4.602) | 103.399(89.451 to 121.165) | 0.944(0.816 to 1.106) | 89.576(77.058 to 104.773) | 0.839(0.721 to 0.981) | 84.274(71.539 to 100.033) | 0.808(0.686 to 0.959) | -4.029  (-4.306 to -3.752) | -0.038(-0.063 to -0.011) |
| 2 to 4 | 151.207(98.178 to 230.715) | 0.408(0.265 to 0.623) | 32.128(26.541 to 39.619) | 0.094(0.077 to 0.115) | 25.174(20.958 to 31.716) | 0.074(0.062 to 0.094) | 22.697(18.362 to 28.72) | 0.068(0.055 to 0.087) | -5.405  (-5.624 to -5.185) | -0.046(-0.069 to -0.021) |
| 5 to 9 | 77.121(48.739 to 117.038) | 0.123(0.078 to 0.187) | 31.942(22.523 to 53.535) | 0.054(0.038 to 0.091) | 28.694(19.811 to 48.937) | 0.049(0.034 to 0.083) | 28.844(19.974 to 48.342) | 0.049(0.034 to 0.082) | -2.296  (-2.622 to -1.968) | -0.044 (-0.067 to -0.019) |
| 10 to 14 | 40.748(26.852 to 59.201) | 0.066(0.044 to 0.096) | 21.686(15.997 to 33.416) | 0.036(0.027 to 0.056) | 21.291(15.502 to 32.649) | 0.036(0.026 to 0.055) | 21.085(15.261 to 32.606) | 0.035(0.025 to 0.054) | -1.488  (-1.82 to -1.155) | -0.026(-0.052 to 0.003) |
| High-middle SDI | | | | | | | | | | |
| 0 to 14 | 34407.118(27556.474 to 41286.667) | 12.575(10.071 to 15.089) | 2252.636(1805.589 to 2734.491) | 0.974(0.78 to 1.182) | 2008.344(1601.775 to 2441.166) | 0.866(0.691 to 1.053) | 1872.697(1455.73 to 2294.971) | 0.811(0.63 to 0.994) | -8.757  (-8.925 to -8.588) | -0.052(-0.076 to -0.027) |
| Neonatal | 2702.65 (2019.147 to 3407.945) | 195.093(145.753 to 246.005) | 272.504(210.955 to 369.207) | 26.909(20.831 to 36.458) | 244.803(186.259 to 339.573) | 26.071(19.836 to 36.164) | 231.295(172.811 to 323.417) | 25.942(19.382 to 36.275) | -7.564  (-7.755 to -7.372) | 0.02(-0.003 to 0.042) |
| 1 to 5 months | 9477.293(7360.078 to 11811.099) | 123.874(96.201 to 154.379) | 701.233 (541.088 to 875.484) | 12.301(9.492 to 15.357) | 654.872(510.656 to 824.146) | 12.366(9.643 to 15.563) | 611.614(470.458 to 774.957) | 12.238(9.414 to 15.506) | -9.499  (-9.813 to -9.184) | 0.015(-0.004 to 0.034) |
| 6 to 11 months | 8905.547(6977.435 to 11015.014) | 98.928(77.51 to 122.362) | 390.52(296.237 to 501.66) | 5.648(4.285 to 7.256) | 340.567(254.152 to 441.263) | 5.286(3.945 to 6.849) | 315.158(228.898 to 420.906) | 5.24(3.806 to 6.998) | -10.346  (-10.588 to -10.103) | -0.002(-0.018 to 0.017) |
| 12 to 23 months | 8031.189(5986.054 to 10354.998) | 43.743(32.604 to 56.4) | 439.696(348.708 to 550.591) | 2.983(2.366 to 3.735) | 383.474(298.985 to 484.743) | 2.78(2.168 to 3.515) | 355.288(270.283 to 450.443) | 2.773(2.109 to 3.515) | -9.3  (-9.533 to -9.066) | -0.026(-0.046 to -0.002) |
| 2 to 4 | 3968.765(2845.288 to 5190.029) | 7.024(5.035 to 9.185) | 221.428(165.427 to 294.17) | 0.459(0.343 to 0.609) | 181.376(134.337 to 243.066) | 0.382(0.283 to 0.512) | 164.774(117.199 to 220.148) | 0.364(0.259 to 0.486) | -9.025  (-9.232 to -8.818) | -0.067(-0.084 to -0.046) |
| 5 to 9 | 874.47(517.464 to 1294.005) | 0.96(0.568 to 1.421) | 132.076(76.263 to 235.573) | 0.168(0.097 to 0.299) | 115.314(65.695 to 208.945) | 0.143(0.082 to 0.26) | 109.479(63.275 to 201.963) | 0.133(0.077 to 0.245) | -5.991  (-6.173 to -5.809) | -0.078(-0.103 to -0.052) |
| 10 to 14 | 447.203(267.335 to 700.464) | 0.499(0.298 to 0.781) | 95.178(54.829 to 156.369) | 0.125(0.072 to 0.206) | 87.938(49.861 to 148.201) | 0.114(0.064 to 0.191) | 85.089(47.23 to 141.089) | 0.108(0.06 to 0.18) | -5.044  (-5.265 to -4.823) | -0.028(-0.059 to 0.003) |
| Middle SDI | | | | | | | | | | |
| 0 to 14 | 304638.576(238427.702 to 360187.622) | 52.777(41.307 to 62.401) | 35309.018(27713.176 to 45251.135) | 6.199(4.866 to 7.945) | 30255.321(23460.748 to 38801.408) | 5.314(4.12 to 6.815) | 28625.018(21973.608 to 37113.689) | 5.05(3.876 to 6.547) | -6.965  (-7.137 to -6.793) | -0.01(-0.027 to 0.005) |
| Neonatal | 28962.106(19014.956 to 38580.227) | 919.853(603.926 to 1225.33) | 2525.815(1890.152 to 3347.784) | 95.858(71.734 to 127.053) | 2222.047(1665.089 to 2962.777) | 88.577(66.375 to 118.104) | 2127.097(1603.747 to 2839.348) | 87.923(66.29 to 117.363) | -7.378  (-7.474 to -7.281) | -0.001(-0.02 to 0.017) |
| 1 to 5 months | 96306.668(74696.247 to 111905.314) | 559.598(434.029 to 650.235) | 10898.522(8381.991 to 13864.615) | 74.177(57.049 to 94.364) | 9228.204(7090.045 to 11850.97) | 65.886(50.62 to 84.612) | 8895.907(6971.872 to 11577.086) | 66.09(51.796 to 86.009) | -7.389  (-7.514 to -7.263) | -0.001(-0.017 to 0.015) |
| 6 to 11 months | 70680.735(56915.359 to 83681.448) | 353.881(284.961 to 418.972) | 8701.78(6538.772 to 11420.922) | 49.534(37.221 to 65.013) | 7573.218(5631.229 to 9949.576) | 45.131(33.558 to 59.292) | 7088.08(5251.859 to 9450.162) | 44.235(32.776 to 58.976) | -6.889  (-7.051 to -6.727) | -0.005(-0.02 to 0.009) |
| 12 to 23 months | 55108.146(42433.947 to 66884.771) | 137.789(106.099 to 167.234) | 6184.845(4642.88 to 8140.697) | 16.904(12.69 to 22.25) | 4811.275(3577.781 to 6441.313) | 13.719(10.202 to 18.367) | 4531.403(3350.865 to 6105.677) | 13.557(10.025 to 18.267) | -6.783  (-7.095 to -6.469) | -0.017(-0.032 to -0.002) |
| 2 to 4 | 37424.411(25979.308 to 46823.046) | 31.133(21.612 to 38.951) | 3395.342(2510.276 to 4684.786) | 2.928(2.165 to 4.04) | 3016.566(2195.773 to 4222.869) | 2.631(1.915 to 3.683) | 2796.915(2027.752 to 3906.865) | 2.513(1.822 to 3.511) | -7.689  (-7.837 to -7.541) | -0.035(-0.054 to -0.019) |
| 5 to 9 | 10574.823(6174.275 to 15570.507) | 5.465(3.191 to 8.047) | 2129.728(1267.478 to 3558.96) | 1.108(0.659 to 1.851) | 2029.547(1131.539 to 3422.522) | 1.044(0.582 to 1.761) | 1890.193(1075.625 to 3152.693) | 0.959(0.546 to 1.6) | -5.152  (-5.283 to -5.02) | -0.027(-0.044 to -0.011) |
| 10 to 14 | 5581.687(3284.693 to 8585.919) | 3.047(1.793 to 4.687) | 1472.988(847.985 to 2408.508) | 0.776(0.447 to 1.269) | 1374.465 (800.303 to 2303.179) | 0.716(0.417 to 1.2) | 1295.425(757.141 to 2093.112) | 0.671(0.392 to 1.084) | -4.565  (-4.726 to -4.404) | 0.003(-0.017 to 0.023) |
| Low-middle SDI | | | | | | | | | | |
| 0 to 14 | 794757.451(647653.807 to 931120.672) | 168.341(137.182 to 197.224) | 125117.12(98855.515 to 161875.123) | 21.506(16.992 to 27.824) | 105945.284(82882.599 to 137738.172) | 18.235(14.265 to 23.707) | 97622.489(76594.921 to 126806.086) | 16.836(13.21 to 21.869) | -6.467  (-6.653 to -6.281) | -0.023 (-0.047 to 0.002) |
| Neonatal | 94516.948(67570.016 to 120342.59) | 3264.715(2333.94 to 4156.76) | 9704.836(7301.79 to 13966.145) | 322.33(242.517 to 463.862) | 8617.253(6328.245 to 12588.987) | 290.596(213.405 to 424.533) | 8030.619(5911.271 to 11746.559) | 273.745(201.501 to 400.413) | -7.392  (-7.507 to -7.277) | -0.066(-0.087 to -0.04) |
| 1 to 5 months | 248093.724(200528.166 to 288428.277) | 1588.486(1283.935 to 1846.738) | 38611.16(29501.781 to 52963.942) | 233.203(178.184 to 319.891) | 30407.237(22080.132 to 42207.782) | 186.32(135.295 to 258.627) | 28429.934(20752.169 to 39843.985) | 176.164(128.59 to 246.891) | -6.231  (-6.409 to -6.053) | -0.074(-0.093 to -0.05) |
| 6 to 11 months | 147847.73(117332.426 to 180557.96) | 822.866(653.029 to 1004.919) | 30461.185(22280.252 to 40438.741) | 156.976(114.817 to 208.393) | 25710.698(18365.808 to 34820.846) | 134.281(95.92 to 181.861) | 23533.085(16987.961 to 32539.31) | 124.436(89.827 to 172.058) | -5.024  (-5.226 to -4.821) | -0.102(-0.12 to -0.082) |
| 12 to 23 months | 126147.138(95321.589 to 153729.048) | 360.311(272.264 to 439.092) | 18370.973(13892.704 to 23969.594) | 47.1(35.618 to 61.454) | 16113.045(12059.484 to 21478.345) | 41.837(31.312 to 55.768) | 14781.824(10993.276 to 19938.8) | 38.864(28.903 to 52.423) | -6.459  (-6.571 to -6.346) | -0.14(-0.161 to -0.118) |
| 2 to 4 | 122725.01(92575.741 to 156238.982) | 120.329(90.768 to 153.188) | 13196.158(9666.599 to 18030.985) | 11.222(8.221 to 15.334) | 11885.684(8551.369 to 16832.639) | 10.189(7.331 to 14.43) | 10726.685(7713.808 to 15228.381) | 9.282(6.675 to 13.178) | -7.678  (-7.943 to -7.412) | -0.119 (-0.153 to -0.088) |
| 5 to 9 | 37612.32(22658.703 to 53149.875) | 23.492(14.152 to 33.196) | 9019.47(5158.278 to 15032.287) | 4.618(2.641 to 7.697) | 7798.786(4300.865 to 13058.971) | 3.995(2.203 to 6.689) | 7036.474(3970.467 to 11456.114) | 3.611(2.038 to 5.879) | -5.307  (-5.607 to -5.006) | -0.016(-0.045 to 0.012) |
| 10 to 14 | 17814.581(11258.051 to 27640.162) | 12.861(8.127 to 19.954) | 5753.339(3408.988 to 9691.707) | 3.014(1.786 to 5.076) | 5412.582(3259.987 to 9007.352) | 2.816(1.696 to 4.687) | 5083.869(2996.506 to 8608.888) | 2.629(1.549 to 4.451) | -4.96  (-5.177 to -4.743) | 0.01(-0.023 to 0.043) |
| Low SDI | | | | | | | | | | |
| 0 to 14 | 602562.58(450215.548 to 752457.754) | 263.227(196.675 to 328.708) | 285213.157(213939.302 to 377664.937) | 63.714(47.792 to 84.367) | 261607.399(189381.236 to 352694.394) | 57.617(41.71 to 77.678) | 245328.66(176547.497 to 335720.04) | 53.306(38.361 to 72.947) | -4.919  (-5.173 to -4.665) | -0.036(-0.056 to -0.015) |
| Neonatal | 47021.847(32072.679 to 61453.07) | 2846.514(1941.552 to 3720.123) | 15890.989(12078.969 to 21840.059) | 597.577(454.227 to 821.291) | 14603.572(10860.812 to 20344.714) | 545.058 (405.365 to 759.338) | 13981.85(10301.986 to 19666.869) | 516.121(380.283 to 725.975) | -5.245  (-5.502 to -4.987) | -0.072(-0.093 to -0.051) |
| 1 to 5 months | 164136.1(125578.865 to 195296.719) | 1859.456(1422.651 to 2212.467) | 74846.878(58424.753 to 95088.098) | 516.376(403.078 to 656.022) | 65967.135(50260.075 to 84822.245) | 451.343(343.876 to 580.348) | 62977.057(47694.514 to 81508.535) | 426.377(322.909 to 551.842) | -4.552  (-4.817 to -4.287) | -0.086(-0.105 to -0.065) |
| 6 to 11 months | 115131.358(83151.608 to 145834.815) | 1154.058(833.498 to 1461.824) | 67162.292(47636.158 to 94163.706) | 401.01(284.424 to 562.229) | 61423.045(41814.178 to 87538.97) | 363.264(247.295 to 517.717) | 57595.031(39107.936 to 83588.862) | 337.555(229.205 to 489.901) | -3.385  (-3.565 to -3.206) | -0.128 (-0.146 to -0.11) |
| 12 to 23 months | 120253.659(85124.628 to 156218.248) | 639.315(452.555 to 830.517) | 54806.782(39077.367 to 75865.785) | 166.517(118.727 to 230.499) | 50747.108(35407.768 to 71009.378) | 152.489(106.396 to 213.374) | 47058.416(32201.506 to 66792.655) | 140.022(95.815 to 198.741) | -4.8  (-5.01 to -4.589) | -0.196 (-0.216 to -0.174) |
| 2 to 4 | 125772.791(82805.405 to 170225.175) | 244.074(160.692 to 330.338) | 52152.883(33404.511 to 76375.54) | 54.783(35.089 to 80.227) | 49527.992(30913.557 to 73982.543) | 51.433(32.103 to 76.828) | 45466.248(27364.43 to 69997.377) | 46.668(28.087 to 71.847) | -5.352  (-5.581 to -5.122) | -0.174 (-0.207 to -0.143) |
| 5 to 9 | 21128.615(10617.637 to 32331.855) | 27.896(14.018 to 42.687) | 13463.388(7508.536 to 22472.508) | 8.988(5.013 to 15.002) | 12664.001(6952.799 to 21418.967) | 8.349(4.584 to 14.12) | 11868.028(6443.283 to 20267.811) | 7.732(4.198 to 13.204) | -3.528  (-3.734 to -3.321) | -0.019(-0.043 to 0.004) |
| 10 to 14 | 9118.208(5080.598 to 13770.161) | 14.618(8.145 to 22.076) | 6889.945(4038.338 to 11744.462) | 5.072(2.973 to 8.646) | 6674.547(3925.417 to 11103.15) | 4.816(2.833 to 8.012) | 6382.03(3630.916 to 10703.466) | 4.521(2.572 to 7.583) | -3.279  (-3.515 to -3.042) | 0.022(-0.003 to 0.052) |
| World Bank High Income | | | | | | | | | | |
| 0 to 14 | 3021.885(2519.652 to 3806.414) | 1.428(1.191 to 1.799) | 858.393 (786.395 to 940.043) | 0.438(0.402 to 0.48) | 755.397(671.533 to 835.814) | 0.388(0.345 to 0.429) | 709.138 (609.328 to 803.443) | 0.366(0.315 to 0.415) | -3.947  (-4.264 to -3.629) | -0.044 (-0.06 to -0.028) |
| Neonatal | 308.327(242.981 to 423.951) | 28.878(22.758 to 39.707) | 127.429(118.183 to 139.14) | 14.138(13.112 to 15.438) | 115.252(103.465 to 127.904) | 13.012(11.682 to 14.441) | 108.219(93.839 to 124.033) | 12.288(10.655 to 14.083) | -2.431  (-2.791 to -2.07) | -0.026(-0.052 to -0.001) |
| 1 to 5 months | 1089.888(901.307 to 1394.501) | 18.503(15.301 to 23.674) | 293.303 (272.065 to 319.153) | 5.846(5.422 to 6.361) | 267.972(239.181 to 295.806) | 5.442(4.857 to 6.007) | 250.548(212.588 to 286.547) | 5.131(4.354 to 5.869) | -4.549  (-4.792 to -4.306) | -0.03(-0.051 to -0.007) |
| 6 to 11 months | 795.743(592.194 to 1076.98) | 11.539(8.587 to 15.617) | 143.99(124.627 to 166.498) | 2.415(2.091 to 2.793) | 115.138(95.519 to 135.798) | 1.973(1.637 to 2.327) | 107.838(86.391 to 128.709) | 1.873(1.501 to 2.236) | -4.951  (-5.463 to -4.436) | -0.036(-0.061 to -0.016) |
| 12 to 23 months | 510.53(396.6 to 685.789) | 3.686(2.864 to 4.952) | 176.838(154.484 to 201.51) | 1.451(1.267 to 1.653) | 154.362(133.5 to 176.402) | 1.295(1.12 to 1.48) | 144.913(121.293 to 170.93) | 1.241(1.039 to 1.464) | -3.141  (-3.455 to -2.826) | -0.045(-0.068 to -0.026) |
| 2 to 4 | 179.075(124.731 to 259.387) | 0.432(0.301 to 0.626) | 47.336(40.784 to 55.555) | 0.124(0.107 to 0.146) | 38.849(33.568 to 45.68) | 0.103(0.089 to 0.122) | 33.611(28.274 to 40.083) | 0.091(0.077 to 0.109) | -4.658  (-4.975 to -4.339) | -0.049(-0.07 to -0.027) |
| 5 to 9 | 90.485(63.962 to 126.154) | 0.128(0.09 to 0.178) | 41.544(31.904 to 62.823) | 0.063(0.048 to 0.095) | 37.227(28.098 to 56.408) | 0.057(0.043 to 0.086) | 37.385(28.036 to 55.892) | 0.057(0.043 to 0.085) | -2.004  (-2.331 to -1.677) | -0.041(-0.062 to -0.02) |
| 10 to 14 | 47.836(35.329 to 64.56) | 0.067(0.049 to 0.09) | 27.951(22.05 to 39.605) | 0.041(0.033 to 0.059) | 26.597(20.593 to 37.891) | 0.039(0.03 to 0.056) | 26.624(20.667 to 37.96) | 0.039(0.03 to 0.056) | -1.072  (-1.403 to -0.739) | -0.025(-0.05 to 0.003) |
| World Bank Upper Middle Income | | | | | | | | | | |
| 0 to 14 | 192157.329(167831.287 to 217000.232) | 31.473(27.489 to 35.542) | 15783.45(13161.534 to 18762.91) | 2.945(2.455 to 3.5) | 13542.46(11162.812 to 16370.423) | 2.521(2.078 to 3.048) | 12682.427(10371.818 to 15327.998) | 2.37(1.938 to 2.864) | -7.798  (-7.999 to -7.597) | -0.039(-0.056 to -0.022) |
| Neonatal | 11503.361(9940.947 to 13208.44) | 351.633(303.873 to 403.753) | 775.43(624.699 to 969.241) | 32.007(25.785 to 40.007) | 681.443(539.558 to 856.077) | 30.325(24.011 to 38.097) | 639.161(500.911 to 800.365) | 29.945(23.468 to 37.497) | -8.427  (-8.674 to -8.179) | 0.023(-0.001 to 0.047) |
| 1 to 5 months | 64832.128(56881.618 to 73169.332) | 361.243(316.943 to 407.698) | 4837.469(3960.262 to 5763.935) | 35.543(29.098 to 42.351) | 4361.956(3574.293 to 5208.528) | 34.475(28.25 to 41.166) | 4116.976(3379.903 to 4974.718) | 34.47(28.299 to 41.652) | -8.486  (-8.71 to -8.26) | 0.017(-0.001 to 0.036) |
| 6 to 11 months | 49069.299(41728.487 to 56997.31) | 234.678(199.57 to 272.594) | 3903.451(3184.463 to 4811.618) | 23.706(19.34 to 29.221) | 3530.517(2808.442 to 4389.028) | 22.976(18.277 to 28.563) | 3286.986(2607.653 to 4077.858) | 22.912(18.177 to 28.425) | -8.374  (-8.652 to -8.096) | 0.003(-0.011 to 0.018) |
| 12 to 23 months | 41537.891(34605.02 to 48899.865) | 98.656(82.19 to 116.142) | 3668.814(2979.648 to 4469.889) | 10.501(8.529 to 12.794) | 2618.905(2070.525 to 3207.762) | 7.991(6.318 to 9.788) | 2423.159(1890.502 to 2995.53) | 7.947(6.2 to 9.824) | -7.415  (-7.821 to -7.007) | -0.023(-0.037 to -0.008) |
| 2 to 4 | 17954.556(14660.732 to 22008.923) | 14.113(11.524 to 17.3) | 1227.511(957.147 to 1539.952) | 1.088(0.848 to 1.365) | 1059.963(798.746 to 1392.657) | 0.951(0.716 to 1.249) | 966.315(722.11 to 1275.025) | 0.904(0.675 to 1.192) | -8.55  (-8.896 to -8.202) | -0.061(-0.076 to -0.048) |
| 5 to 9 | 5013.235(3469.652 to 6536.193) | 2.474(1.712 to 3.226) | 867.847(632.936 to 1258.826) | 0.48(0.35 to 0.696) | 794.379(566.38 to 1160.111) | 0.43(0.307 to 0.628) | 764.946(538.335 to 1138.796) | 0.402(0.283 to 0.599) | -5.484  (-5.705 to -5.263) | -0.065(-0.081 to -0.049) |
| 10 to 14 | 2246.859(1592.848 to 3164.598) | 1.144(0.811 to 1.611) | 502.928(350.75 to 707.016) | 0.287(0.2 to 0.404) | 495.297(342.281 to 701.558) | 0.279(0.193 to 0.395) | 484.884(332.141 to 697.574) | 0.27(0.185 to 0.389) | -4.499  (-4.813 to -4.183) | -0.015(-0.034 to 0.005) |
| World Bank Lower Middle Income | | | | | | | | | | |
| 0 to 14 | 1203982.15(946751.783 to 1417065.41) | 157.796(124.083 to 185.723) | 284748.384(223042.59 to 360166.562) | 29.106(22.798 to 36.815) | 247622.66(191844.926 to 323520.598) | 25.295(19.598 to 33.049) | 230353.301(176087.552 to 302402.045) | 23.548(18.001 to 30.913) | -5.175  (-5.345 to -5.004) | -0.02(-0.036 to -0.003) |
| Neonatal | 138187.331(93222.422 to 173573.632) | 3029.167(2043.503 to 3804.86) | 18946.457(14600.362 to 25279.07) | 377.499(290.905 to 503.673) | 16798.97(12772.657 to 23043.856) | 339.122(257.843 to 465.188) | 15791.51(11922.213 to 21904.57) | 321.865(243.001 to 446.463) | -6.515  (-6.627 to -6.404) | -0.066(-0.082 to -0.049) |
| 1 to 5 months | 353117.519(282723.017 to 411726.004) | 1430.762(1145.537 to 1668.232) | 80549.771(64188.714 to 104612.264) | 291.92(232.626 to 379.124) | 65010.699(51123.65 to 83711.246) | 238.539(187.585 to 307.156) | 61470.221(48220.318 to 80349.527) | 227.824(178.716 to 297.795) | -5.165  (-5.302 to -5.029) | -0.073(-0.087 to -0.059) |
| 6 to 11 months | 222974.087(175734.976 to 269070.47) | 783.473 (617.487 to 945.443) | 68210.893(52449.134 to 88982.793) | 211.055(162.286 to 275.327) | 58791.888(43748.377 to 78988.021) | 184.009(136.925 to 247.22) | 54714.092(40645.265 to 75149.814) | 173.17(128.642 to 237.849) | -3.908  (-4.026 to -3.791) | -0.096(-0.108 to -0.082) |
| 12 to 23 months | 202558.225(148857.046 to 249763.991) | 363.027(266.783 to 447.63) | 49620.038(36411.745 to 64190.516) | 76.321(56.005 to 98.732) | 44486.4(32008.473 to 59503.809) | 69.155(49.758 to 92.5) | 41212.706(29331.495 to 56256.119) | 64.801(46.119 to 88.454) | -4.822  (-4.949 to -4.695) | -0.123 (-0.138 to -0.107) |
| 2 to 4 | 203329.282(147603.526 to 256526.823) | 123.88(89.929 to 156.291) | 41063.566(27843.108 to 56528.284) | 20.845(14.134 to 28.696) | 38604.127(25056.785 to 54486.441) | 19.735(12.809 to 27.854) | 35168.364(22708.096 to 51397.717) | 18.136(11.711 to 26.506) | -5.535  (-5.753 to -5.316) | -0.098(-0.122 to -0.075) |
| 5 to 9 | 56853.337(32269.066 to 81399.865) | 21.872(12.414 to 31.316) | 16294.293(9484.314 to 27655.941) | 4.934(2.872 to 8.375) | 14459.229(8309.463 to 24739.252) | 4.375(2.514 to 7.485) | 13134.593(7495.747 to 22084.928) | 3.979(2.271 to 6.691) | -4.69  (-4.97 to -4.409) | -0.012(-0.03 to 0.005) |
| 10 to 14 | 26962.369(16985.121 to 42077.571) | 11.96(7.534 to 18.665) | 10063.366(6003.509 to 16885.988) | 3.133(1.869 to 5.258) | 9471.347(5673.294 to 15860.415) | 2.92(1.749 to 4.891) | 8861.815(5150.236 to 15064.649) | 2.709(1.574 to 4.605) | -4.355  (-4.556 to -4.153) | 0.009(-0.011 to 0.029) |
| World Bank Low Income | | | | | | | | | | |
| 0 to 14 | 339411.458(250943.227 to 432800.253) | 222.593(164.574 to 283.839) | 147073.132(106728.624 to 205322.536) | 49.986(36.274 to 69.783) | 138400.043(99006.934 to 197549.797) | 46.306(33.126 to 66.096) | 130178.404(91870.18 to 186314.75) | 42.901(30.276 to 61.401) | -5.539  (-5.828 to -5.25) | -0.037(-0.062 to -0.016) |
| Neonatal | 23445.652(17034.877 to 30354.257) | 2073.974(1506.884 to 2685.101) | 8638.436(6085.199 to 12708.686) | 484.919(341.593 to 713.403) | 8176.687(5642.697 to 12096.04) | 455.44(314.297 to 673.747) | 7911.629(5399.649 to 11703.202) | 435.687(297.354 to 644.485) | -5.264  (-5.598 to -4.929) | -0.055(-0.08 to -0.03) |
| 1 to 5 months | 99745.506(72824.087 to 121189.878) | 1653.163(1206.972 to 2008.577) | 39576.248(29793.491 to 52331.504) | 407.258(306.589 to 538.516) | 36800.69(27315.924 to 48749.384) | 375.484(278.709 to 497.399) | 35248.837(26226.816 to 47209.833) | 355.834(264.757 to 476.579) | -5.389  (-5.745 to -5.032) | -0.069(-0.093 to -0.047) |
| 6 to 11 months | 70266.049(49296.614 to 91606.202) | 1034.03(725.446 to 1348.07) | 34547.131(23058.435 to 51432.465) | 307.545(205.271 to 457.862) | 32680.994(21198.545 to 49208.005) | 288.148(186.907 to 433.867) | 30488.297(19518.674 to 46608.102) | 266.426(170.566 to 407.291) | -4.179  (-4.428 to -3.93) | -0.118 (-0.14 to -0.094) |
| 12 to 23 months | 65319.357(46153.495 to 86853.85) | 511.704(361.561 to 680.403) | 26439.988(18169.018 to 38433.049) | 119.843(82.354 to 174.204) | 24884.77(16656.56 to 36789.911) | 111.494(74.629 to 164.835) | 23030.386(14800.06 to 34645.341) | 102.159(65.65 to 153.681) | -5.515  (-5.768 to -5.262) | -0.214 (-0.24 to -0.188) |
| 2 to 4 | 68579.256(44492.703 to 95139.77) | 199.099(129.171 to 276.21) | 26659.524(16897.423 to 40891.497) | 42.051(26.653 to 64.5) | 24933.83(15326.581 to 39180.232) | 38.797(23.848 to 60.964) | 23009.006(13817.255 to 36813.524) | 35.335(21.219 to 56.535) | -6.02  (-6.218 to -5.821) | -0.206 (-0.241 to -0.174) |
| 5 to 9 | 8310.283(4011.521 to 14023.834) | 16.721(8.072 to 28.217) | 7572.917(3988.643 to 12222.73) | 7.743(4.078 to 12.497) | 7345.497(3776.982 to 12272.055) | 7.392(3.801 to 12.349) | 6996.084(3612.984 to 11841.813) | 6.932(3.58 to 11.734) | -2.486  (-2.644 to -2.327) | -0.018(-0.049 to 0.009) |
| 10 to 14 | 3745.354(1892.572 to 6189.868) | 9.001(4.548 to 14.875) | 3638.889(2050.36 to 6080.167) | 4.124(2.324 to 6.891) | 3577.574(1970.665 to 5851.031) | 3.976(2.19 to 6.502) | 3494.166(1925.225 to 5871.288) | 3.811(2.1 to 6.403) | -2.595  (-2.758 to -2.432) | 0.03(-0.003 to 0.059) |
| North Africa and Middle East | | | | | | | | | | |
| 0 to 14 | 91953.216(69091.57 to 117564.012) | 65.454(49.181 to 83.684) | 15431.41(11227.372 to 22585.974) | 8.468(6.161 to 12.393) | 13127.528(9400.637 to 19643.202) | 7.174(5.137 to 10.734) | 12021.149(8527.802 to 18090.537) | 6.557(4.652 to 9.868) | -6.614  (-6.898 to -6.33) | -0.086(-0.123 to -0.053) |
| Neonatal | 12388.491(8802.387 to 17390.484) | 1505.227(1069.508 to 2112.98) | 1746.354(1156.542 to 3044.189) | 183.601(121.592 to 320.048) | 1516.185(970.479 to 2681.657) | 163.968(104.952 to 290.008) | 1418.454(914.455 to 2493.968) | 156.913(101.159 to 275.889) | -7.253  (-7.611 to -6.894) | -0.02(-0.071 to 0.032) |
| 1 to 5 months | 30687.043(22922.826 to 40076.397) | 685.051(511.724 to 894.657) | 4852.594(3531.15 to 6982.775) | 92.064(66.994 to 132.478) | 4071.246(2867.414 to 5957.008) | 79.414(55.932 to 116.199) | 3772.479(2624.281 to 5690.202) | 75.355(52.42 to 113.662) | -7.871  (-8.245 to -7.495) | -0.04(-0.087 to 0.009) |
| 6 to 11 months | 22782.66(16837.57 to 29347.916) | 438.693(324.217 to 565.11) | 4341.95(2903.628 to 6334.783) | 69.611(46.552 to 101.561) | 3669.261(2331.33 to 5630.717) | 60.459(38.414 to 92.778) | 3283.211(2062.409 to 5085.562) | 55.518(34.875 to 85.995) | -5.165  (-5.493 to -4.835) | -0.111(-0.146 to -0.072) |
| 12 to 23 months | 15007.431(10604.47 to 19987.011) | 145.993(103.161 to 194.435) | 2607.156(1778.736 to 3904.796) | 20.609(14.061 to 30.867) | 2248.048(1541.097 to 3397.918) | 18.223(12.493 to 27.544) | 2045.113(1383.289 to 3100.041) | 17.031(11.519 to 25.816) | -6.347  (-6.613 to -6.081) | -0.278(-0.317 to -0.238) |
| 2 to 4 | 9518.935(6419.27 to 13532.214) | 31.257(21.078 to 44.435) | 1258.172 (805.363 to 2064.943) | 3.26(2.087 to 5.35) | 1056.505(648.226 to 1774.712) | 2.777(1.704 to 4.665) | 954.227(584.476 to 1563.219) | 2.558(1.567 to 4.19) | -7.708  (-8.219 to -7.194) | -0.266(-0.318 to -0.219) |
| 5 to 9 | 1076.556(424.976 to 1744.373) | 2.267(0.895 to 3.673) | 408.243 (212.254 to 693.422) | 0.657(0.342 to 1.117) | 363.396(174.493 to 639.176) | 0.578(0.278 to 1.017) | 347.958(169.718 to 615.3) | 0.551(0.269 to 0.974) | -4.081  (-4.194 to -3.968) | -0.059(-0.099 to -0.019) |
| 10 to 14 | 492.101(241.495 to 783.344) | 1.178(0.578 to 1.876) | 216.941(106.582 to 375.93) | 0.384(0.189 to 0.666) | 202.888(93.565 to 360.86) | 0.352(0.162 to 0.626) | 199.709(92.608 to 352.523) | 0.339(0.157 to 0.598) | -3.854  (-3.939 to -3.77) | 0.009(-0.027 to 0.051) |
| South Asia | | | | | | | | | | |
| 0 to 14 | 679060.009(551103.007 to 817209.497) | 156.696(127.169 to 188.574) | 93626.437(65839.588 to 130178.792) | 18.192(12.793 to 25.295) | 77620.341(52694.331 to 108080.89) | 15.187(10.31 to 21.147) | 69139.936(46159.464 to 95752.928) | 13.636(9.104 to 18.885) | -6.82  (-6.995 to -6.645) | -0.005(-0.028 to 0.018) |
| Neonatal | 89771.771(63463.452 to 114129.993) | 3501.999(2475.711 to 4452.214) | 7875.157(5188.797 to 12018.1) | 320.956(211.472 to 489.803) | 7205.139(4552.809 to 11054.015) | 298.696(188.741 to 458.255) | 6505.762(4065.343 to 9947.821) | 273.52(170.918 to 418.234) | -7.687  (-7.841 to -7.533) | -0.102(-0.128 to -0.072) |
| 1 to 5 months | 203510.287(163983.085 to 241206.637) | 1470.963(1185.262 to 1743.43) | 28733.197(17258.308 to 42972.058) | 212.941(127.901 to 318.465) | 20915.675(12253.534 to 30731.217) | 157.52(92.284 to 231.443) | 18789.772(10897.874 to 28225.935) | 143.517(83.238 to 215.59) | -6.132  (-6.298 to -5.966) | -0.104 (-0.129 to -0.079) |
| 6 to 11 months | 106754.935(80660.648 to 134787.492) | 668.939(505.429 to 844.595) | 20064.949(12106.075 to 29574.072) | 126.759(76.479 to 186.832) | 16249.52(9173.605 to 24067.005) | 104.186(58.818 to 154.309) | 14498.031(8121.055 to 22050.099) | 94.324(52.836 to 143.458) | -4.978  (-5.147 to -4.808) | -0.115(-0.138 to -0.089) |
| 12 to 23 months | 101994.385(74733.248 to 130384.798) | 325.4(238.427 to 415.976) | 11152.258(7134.925 to 15817.886) | 34.737(22.224 to 49.27) | 10148.184(6318.622 to 14724.242) | 32.074(19.971 to 46.537) | 9022.93(5568.971 to 12731.633) | 28.949(17.867 to 40.847) | -7.314  (-7.433 to -7.194) | -0.117 (-0.151 to -0.079) |
| 2 to 4 | 111445.974(82017.905 to 144577.609) | 119.418(87.885 to 154.92) | 9055.998(5627.521 to 13522.149) | 9.135(5.677 to 13.641) | 8389.275(4921.01 to 12429.428) | 8.569(5.026 to 12.696) | 7344.293(4290.907 to 11134.737) | 7.604(4.443 to 11.529) | -8.577  (-8.885 to -8.267) | -0.06(-0.102 to -0.018) |
| 5 to 9 | 44735.344(25893.83 to 64411.625) | 29.917(17.317 to 43.076) | 9761.176(5313.93 to 16430.688) | 5.631(3.065 to 9.478) | 8221.828(4262.966 to 13643.298) | 4.781(2.479 to 7.934) | 7043.996(3683.13 to 11361.644) | 4.136(2.162 to 6.67) | -5.444  (-5.817 to -5.068) | 0(-0.025 to 0.026) |
| 10 to 14 | 20847.312(12852.102 to 31900.647) | 16.44(10.135 to 25.157) | 6983.702(4108.297 to 12005.811) | 3.917(2.305 to 6.735) | 6490.72(3836.084 to 10949.9) | 3.64(2.151 to 6.141) | 5935.153(3371.967 to 10056.506) | 3.332(1.893 to 5.647) | -4.859  (-5.125 to -4.592) | 0.011(-0.015 to 0.04) |
| High-income | | | | | | | | | | |
| 0 to 14 | 1495.411(1405.479 to 1590.15) | 0.798(0.75 to 0.848) | 526.256(497.837 to 560.424) | 0.294(0.278 to 0.313) | 458.523(418.747 to 500.939) | 0.258(0.236 to 0.282) | 427.101(373.038 to 484.923) | 0.242(0.211 to 0.275) | -2.785  (-3.044 to -2.526) | -0.053(-0.071 to -0.037) |
| Neonatal | 130.733(122.761 to 140.765) | 13.708(12.873 to 14.76) | 85.992(80.883 to 92.12) | 10.555(9.928 to 11.307) | 78.164(70.568 to 85.848) | 9.766(8.817 to 10.726) | 73.182(62.656 to 83.71) | 9.176(7.856 to 10.496) | -0.341  (-0.585 to -0.096) | -0.025(-0.054 to 0.005) |
| 1 to 5 months | 694.436(639.093 to 753.221) | 13.206(12.154 to 14.324) | 188.616(174.571 to 204.305) | 4.159(3.849 to 4.505) | 168.325(152.742 to 186.77) | 3.786(3.435 to 4.2) | 158.508(136.339 to 182.175) | 3.588(3.086 to 4.124) | -3.928  (-4.172 to -3.682) | -0.029(-0.053 to -0.003) |
| 6 to 11 months | 259.679(232.647 to 287.32) | 4.222(3.783 to 4.672) | 72.659(64.793 to 81.371) | 1.348(1.202 to 1.51) | 56.781(48.558 to 66.081) | 1.079(0.923 to 1.255) | 51.298(42.728 to 60.945) | 0.986(0.822 to 1.172) | -3.177  (-3.507 to -2.845) | -0.035(-0.062 to -0.012) |
| 12 to 23 months | 303.429(276.67 to 332.496) | 2.458(2.241 to 2.694) | 108.247(98.561 to 118.847) | 0.981(0.893 to 1.077) | 92.469(82.712 to 102.828) | 0.859(0.768 to 0.955) | 84.203(72.825 to 96.869) | 0.799(0.691 to 0.92) | -2.381  (-2.673 to -2.089) | -0.044 (-0.069 to -0.02) |
| 2 to 4 | 50.841(46.598 to 55.138) | 0.138(0.126 to 0.15) | 28.081(26.124 to 30.069) | 0.081(0.075 to 0.087) | 23.636(21.244 to 25.841) | 0.069(0.062 to 0.076) | 19.975(17.767 to 22.25) | 0.06(0.053 to 0.067) | -1.069  (-1.435 to -0.702) | -0.05(-0.073 to -0.025) |
| 5 to 9 | 36.134(28.559 to 45.867) | 0.058(0.046 to 0.073) | 25.596(23.518 to 27.991) | 0.042(0.039 to 0.046) | 22.73(20.761 to 24.816) | 0.038(0.035 to 0.041) | 23.546(21.261 to 25.971) | 0.039(0.036 to 0.043) | -0.183  (-0.607 to 0.242) | -0.055(-0.078 to -0.034) |
| 10 to 14 | 20.16(15.991 to 24.808) | 0.032(0.025 to 0.039) | 17.065(15.792 to 18.597) | 0.027(0.025 to 0.03) | 16.418(14.998 to 17.977) | 0.026(0.024 to 0.029) | 16.39(14.902 to 18.065) | 0.026(0.024 to 0.029) | 0.344  (-0.105 to 0.795) | -0.042(-0.069 to -0.012) |
| Central Europe, Eastern Europe, and Central Asia | | | | | | | | | | |
| 0 to 14 | 16048.808(14640.475 to 17683.295) | 15.149(13.82 to 16.692) | 2261.238(1708.928 to 2920.481) | 2.806(2.121 to 3.624) | 2095.225(1588.328 to 2757.971) | 2.592(1.965 to 3.412) | 2049.79(1545.178 to 2682.207) | 2.536(1.912 to 3.319) | -6.236  (-6.533 to -5.938) | -0.14 (-0.167 to -0.112) |
| Neonatal | 1091.198(989.077 to 1211.703) | 215.691(195.506 to 239.511) | 188.55(142.855 to 253.595) | 48.276(36.576 to 64.93) | 176.629(132.635 to 239.876) | 46.332(34.792 to 62.922) | 171.863(127.025 to 236.995) | 46.258(34.189 to 63.788) | -6.239  (-6.692 to -5.784) | -0.051(-0.074 to -0.026) |
| 1 to 5 months | 4326.734(3838.112 to 4860.66) | 153.515(136.179 to 172.459) | 604.143 (438.513 to 823.936) | 27.83(20.2 to 37.955) | 574.292(414.027 to 796.035) | 27.106(19.541 to 37.572) | 556.488(397.752 to 776.502) | 26.937(19.253 to 37.587) | -7.834  (-8.441 to -7.223) | -0.064 (-0.084 to -0.043) |
| 6 to 11 months | 6780.327(6084.79 to 7574.012) | 201.257(180.612 to 224.816) | 906.898(664.81 to 1193.573) | 35.189(25.796 to 46.313) | 828.389(585.211 to 1108.445) | 32.945(23.274 to 44.083) | 809.568(569.63 to 1076.989) | 32.992(23.214 to 43.889) | -6.902  (-7.406 to -6.394) | -0.106 (-0.123 to -0.087) |
| 12 to 23 months | 2698.918(2383.508 to 3071.955) | 38.477(33.981 to 43.796) | 345.181(254.952 to 465.397) | 6.549(4.837 to 8.83) | 320.403(229.945 to 449.962) | 6.247(4.483 to 8.773) | 316.754(226.956 to 443.038) | 6.329(4.534 to 8.852) | -8.342  (-9.054 to -7.625) | -0.18(-0.201 to -0.16) |
| 2 to 4 | 936.579(785.2 to 1101.322) | 4.221(3.538 to 4.963) | 156.828(109.261 to 225.205) | 0.944(0.658 to 1.356) | 138.482(93.248 to 206.691) | 0.853(0.574 to 1.273) | 137.675(91.118 to 210.481) | 0.871(0.577 to 1.332) | -6.901  (-7.487 to -6.312) | -0.226 (-0.244 to -0.21) |
| 5 to 9 | 152.204(130.712 to 172.573) | 0.425(0.365 to 0.482) | 41.156(26.883 to 59.756) | 0.147(0.096 to 0.213) | 39.239(25.033 to 58.338) | 0.139(0.089 to 0.207) | 39.381(24.567 to 60.302) | 0.14(0.087 to 0.214) | -5.293  (-5.93 to -4.651) | -0.15(-0.179 to -0.122) |
| 10 to 14 | 62.849(54.844 to 70.107) | 0.184(0.16 to 0.205) | 18.482(13.233 to 24.753) | 0.072(0.052 to 0.097) | 17.79(12.529 to 24.349) | 0.068(0.048 to 0.093) | 18.059(12.605 to 24.935) | 0.067(0.047 to 0.092) | -4.521  (-4.985 to -4.055) | -0.072(-0.101 to -0.044) |
| Southeast Asia, East Asia, and Oceania | | | | | | | | | | |
| 0 to 14 | 257241.981(178710.822 to 322570.717) | 51.115(35.511 to 64.096) | 20523.036(15670.645 to 26355.241) | 4.619(3.527 to 5.931) | 18928.779(14478.759 to 24413.877) | 4.244(3.246 to 5.473) | 17810.68(13561.533 to 22762.359) | 4.002(3.047 to 5.114) | -7.683  (-7.827 to -7.538) | -0.025(-0.042 to -0.007) |
| Neonatal | 24777.542(12586.765 to 36759.828) | 899.16(456.765 to 1333.989) | 1633.959(1198.863 to 2307.536) | 81.698(59.943 to 115.376) | 1493.576(1112.611 to 2079.906) | 80.727(60.136 to 112.418) | 1435.665(1060.168 to 1980.048) | 81.595(60.254 to 112.535) | -8.103  (-8.248 to -7.957) | 0.092(0.065 to 0.113) |
| 1 to 5 months | 77857.321(54029.375 to 98214.061) | 516.564(358.472 to 651.626) | 6620.045(4832.052 to 8702.675) | 58.885(42.981 to 77.41) | 6191.855(4597.807 to 8198.114) | 59.374(44.089 to 78.612) | 5867.969(4399.716 to 7778.86) | 59.614(44.698 to 79.028) | -7.999  (-8.261 to -7.737) | 0.092(0.069 to 0.109) |
| 6 to 11 months | 58780.128(42864.788 to 73326.687) | 336.136 (245.124 to 419.321) | 4776.81(3476.06 to 6489.167) | 35.068(25.519 to 47.638) | 4415.786(3204.001 to 5957.655) | 34.782(25.237 to 46.926) | 4086.968(2898.71 to 5558.325) | 34.56(24.512 to 47.002) | -7.649  (-7.762 to -7.535) | 0.087(0.066 to 0.105) |
| 12 to 23 months | 45849.16(31747.14 to 58153.429) | 131.043(90.737 to 166.21) | 3612.949(2653.137 to 4902.224) | 12.448(9.141 to 16.89) | 3303.446(2387.87 to 4502.495) | 12.14(8.775 to 16.546) | 3113.555(2250.643 to 4251.461) | 12.326(8.91 to 16.831) | -7.763  (-7.942 to -7.584) | 0.065(0.046 to 0.086) |
| 2 to 4 | 37631.406(23202.537 to 50256.277) | 35.928(22.153 to 47.982) | 2126.979(1544.206 to 2909.317) | 2.266(1.645 to 3.1) | 1873.535(1348.494 to 2528.265) | 2.009(1.446 to 2.71) | 1755.029(1247.422 to 2398.706) | 1.959(1.392 to 2.677) | -8.933  (-9.013 to -8.853) | -0.01(-0.028 to 0.011) |
| 5 to 9 | 7927.724(3955.38 to 13757.81) | 4.755(2.372 to 8.252) | 1065.137(646.099 to 1902.274) | 0.713(0.433 to 1.274) | 1002.822 (602.525 to 1763.807) | 0.655(0.394 to 1.152) | 954.09(576.946 to 1685.539) | 0.602(0.364 to 1.064) | -5.708  (-5.893 to -5.523) | -0.059(-0.079 to -0.036) |
| 10 to 14 | 4418.7(2311.083 to 8155.375) | 2.736(1.431 to 5.05) | 687.158(424.814 to 1203.932) | 0.473(0.293 to 0.829) | 647.76(393.682 to 1147.138) | 0.439(0.267 to 0.778) | 597.402(362.896 to 1060.456) | 0.403(0.245 to 0.714) | -5.164  (-5.574 to -4.752) | -0.019(-0.042 to 0.006) |
| Latin America and Caribbean | | | | | | | | | | |
| 0 to 14 | 94972.317(87666.942 to 102666.872) | 65.834(60.77 to 71.168) | 10182.003(8091.022 to 12723.912) | 7.045(5.599 to 8.804) | 9225.278(7022.359 to 11708.52) | 6.41(4.879 to 8.135) | 8760.027(6524.792 to 11263.551) | 6.114(4.554 to 7.861) | -7.401  (-7.77 to -7.032) | -0.013 (-0.031 to 0.007) |
| Neonatal | 6508.256(5876.499 to 7283.157) | 827.486 (747.162 to 926.01) | 641.43(439.86 to 879.008) | 86.84(59.55 to 119.005) | 597.611(401.479 to 834.614) | 82.552(55.459 to 115.291) | 578.788(383.659 to 819.208) | 81.339(53.917 to 115.125) | -7.151  (-7.841 to -6.455) | -0.015(-0.04 to 0.014) |
| 1 to 5 months | 38556.368(35238.3 to 41944.195) | 897.361 (820.136 to 976.209) | 3029.56(2230.081 to 3915.351) | 74.137(54.573 to 95.813) | 2738.907(1969.779 to 3644.072) | 68.365(49.167 to 90.959) | 2636.704(1887.23 to 3586.938) | 66.988(47.947 to 91.129) | -8.398  (-8.722 to -8.073) | -0.025(-0.042 to -0.006) |
| 6 to 11 months | 21816.152(19620.364 to 24208.061) | 437.861(393.79 to 485.868) | 2459.89(1790.135 to 3291.545) | 51.048(37.149 to 68.307) | 2352.285(1635.566 to 3172.265) | 49.784(34.615 to 67.138) | 2218.634(1544.118 to 3038.815) | 47.841(33.296 to 65.527) | -7.222  (-7.501 to -6.942) | -0.036(-0.054 to -0.018) |
| 12 to 23 months | 17680.032(16168.797 to 19363.83) | 178.185(162.954 to 195.154) | 2510.714(1949.205 to 3140.949) | 25.831(20.054 to 32.315) | 2208.679(1668.23 to 2878.77) | 23.134(17.473 to 30.152) | 2092.061(1529.761 to 2736.441) | 22.32(16.321 to 29.195) | -6.194  (-6.512 to -5.874) | -0.062(-0.084 to -0.039) |
| 2 to 4 | 7157.857(6247.655 to 8065.53) | 24.246(21.163 to 27.32) | 869.839 (638.328 to 1194.529) | 2.967(2.178 to 4.075) | 722.626(506.813 to 1036.985) | 2.489(1.746 to 3.572) | 663.26(454.773 to 959.847) | 2.314(1.587 to 3.349) | -7.126  (-7.496 to -6.755) | -0.062(-0.087 to -0.039) |
| 5 to 9 | 2304.879(2041.607 to 2586.001) | 4.749(4.207 to 5.329) | 435.976(348.257 to 556.801) | 0.905(0.723 to 1.155) | 389.233(297.34 to 505.27) | 0.807(0.617 to 1.048) | 362.572(276.469 to 471.094) | 0.753(0.574 to 0.978) | -5.176  (-5.724 to -4.625) | -0.002(-0.024 to 0.021) |
| 10 to 14 | 948.773(843.427 to 1065.249) | 2.053(1.825 to 2.305) | 234.595(194.649 to 286.689) | 0.492(0.409 to 0.602) | 215.937(175.741 to 268.596) | 0.453(0.369 to 0.563) | 208.008(166.974 to 263.121) | 0.435(0.349 to 0.551) | -4.638  (-5.207 to -4.066) | 0.022(-0.007 to 0.052) |
| Sub-Saharan Africa | | | | | | | | | | |
| 0 to 14 | 598844.611(419893.07 to 750169.711) | 266.819 (187.086 to 334.243) | 306262.137(224212.497 to 419556.91) | 66.482(48.671 to 91.076) | 279199.644(198260.249 to 389673.76) | 59.591(42.316 to 83.17) | 264036.939(186466.383 to 372856.373) | 55.477(39.178 to 78.341) | -4.768  (-5.095 to -4.44) | -0.03(-0.052 to -0.011) |
| Neonatal | 38872.298(24120.222 to 51075.237) | 2353.924(1460.607 to 3092.876) | 16342.112(12266.579 to 22115.074) | 587.072(440.663 to 794.459) | 14729.519(10880.561 to 20306.229) | 526.569(388.972 to 725.932) | 14290.738(10429.957 to 19758.984) | 506.312 (369.527 to 700.048) | -4.5  (-4.841 to -4.159) | -0.062(-0.078 to -0.046) |
| 1 to 5 months | 163479.704(119598.419 to 196037.893) | 1850.299(1353.641 to 2218.8) | 81330.515(62828.154 to 105265.387) | 535.653(413.794 to 693.291) | 71877.541(53997.728 to 93406.738) | 470.709(353.618 to 611.698) | 69398.571(52100.837 to 90870.118) | 450.771(338.415 to 590.237) | -4.369  (-4.714 to -4.023) | -0.077(-0.092 to -0.062) |
| 6 to 11 months | 126191.324(90920.17 to 159831.328) | 1265.497(911.784 to 1602.852) | 74270.925(51774.12 to 107817.914) | 422.993(294.868 to 614.053) | 67631.749(45055.078 to 99676.577) | 382.527(254.833 to 563.774) | 63731.123(42991.686 to 94681.87) | 358.22(241.648 to 532.188) | -3.619  (-3.87 to -3.368) | -0.123 (-0.139 to -0.106) |
| 12 to 23 months | 126589.999(84560.995 to 163048.066) | 676.035(451.585 to 870.734) | 59649.44(41501.884 to 83913.32) | 172.913(120.307 to 243.25) | 53901.159(36339.853 to 78350.438) | 154.848(104.397 to 225.086) | 50211.383(33182.85 to 73735.462) | 143.202(94.637 to 210.292) | -4.821  (-5.074 to -4.567) | -0.188(-0.207 to -0.167) |
| 2 to 4 | 123432.039(77616.722 to 167724.338) | 244.399(153.683 to 332.099) | 55537.831(34956.357 to 83720.546) | 55.842(35.148 to 84.179) | 52466.676(31421.022 to 80034.665) | 52.119(31.213 to 79.505) | 48334.992(28398.75 to 75658.705) | 47.491(27.903 to 74.337) | -5.242  (-5.524 to -4.959) | -0.172(-0.199 to -0.146) |
| 5 to 9 | 14055.699(7382.988 to 23006.324) | 19.256(10.114 to 31.518) | 13050.255(7219.526 to 21863.525) | 8.476(4.689 to 14.2) | 12607.947(6805.119 to 21533.314) | 8.051(4.345 to 13.75) | 12171.819(6583.071 to 20704.083) | 7.651(4.138 to 13.014) | -2.552  (-2.739 to -2.364) | -0.015(-0.041 to 0.007) |
| 10 to 14 | 6223.548(3361.307 to 10335.717) | 10.078(5.443 to 16.736) | 6081.058(3429.453 to 10338.059) | 4.431(2.499 to 7.534) | 5985.054(3448.218 to 9931.306) | 4.254(2.451 to 7.059) | 5898.314(3356.393 to 9838.493) | 4.096(2.331 to 6.832) | -2.57  (-2.762 to -2.377) | 0.032(0.009 to 0.052) |

Values in parentheses are 95% uncertainty intervals. Count data are presented to three significant figures. GBD=Global Burden of Diseases, Injuries, and Risk Factors Study. SDI=Socio-demographic Index.

Supplementary Table 2 Number of deaths, mortality rates and annual percentage change of mortality rates of children aged 0-14 years with diarrhea from 1990 to 2021

| location | 1990 | | 2019 | | 2020 | | 2021 | | Death rate change, % | |
| --- | --- | --- | --- | --- | --- | --- | --- | --- | --- | --- |
|  | Death counts | Death rate per  100 000  population | Death counts | Death rate per  100 000  population | Death counts | Death rate per  100 000  population | Death counts | Death rate per  100 000  population | 1990-2019 | 2019-2021 |
| Global | | | | | | | | | | |
| 0 to 14 | 1739616.352(1373131.016 to 2048270.642) | 100.027(78.954 to 117.774) | 448812.517(346906.545 to 575140.621) | 22.374(17.294 to 28.672) | 400655.318(305575.673 to 525359.77) | 19.921(15.194 to 26.121) | 374245.623(281541.312 to 498683.54) | 18.602(13.994 to 24.787) | -4.704  (-4.926 to -4.482) | -0.169 (-0.22 to -0.113) |
| Neonatal | 173540.288(121913.402 to 213654.379) | 1728.563(1214.329 to 2128.123) | 28513.553(21872.155 to 38532.437) | 281.41(215.864 to 380.29) | 25796.823(19479.199 to 35511.495) | 260.843(196.963 to 359.073) | 24474.451(18314.396 to 34191.137) | 251.143(187.932 to 350.851) | -5.911  (-6.096 to -5.726) | -0.108(-0.17 to -0.055) |
| 1 to 5 months | 519111.891(420211.626 to 598302.318) | 950.829(769.679 to 1095.878) | 125358.67(99381.657 to 159257.145) | 223.924(177.522 to 284.476) | 106537.843(83491.517 to 135449.079) | 194.858(152.706 to 247.736) | 101180.492(78489.364 to 129893.636) | 188.218(146.008 to 241.631) | -5.021  (-5.256 to -4.785) | -0.159(-0.21 to -0.108) |
| 6 to 11 months | 343365.205(270491.54 to 410049.272) | 544.054(428.587 to 649.713) | 106894.081(79842.013 to 143958.109) | 161.884(120.915 to 218.015) | 95203.771(69664.723 to 131485.142) | 147.501(107.933 to 203.712) | 88678.832(63716.955 to 124323.263) | 140.332(100.83 to 196.738) | -3.836  (-4.027 to -3.646) | -0.133 (-0.203 to -0.067) |
| 12 to 23 months | 310123.354(233713.307 to 381694.218) | 248.859(187.544 to 306.291) | 79985.945(58975.852 to 105260.729) | 59.555(43.911 to 78.374) | 72222.388(52188.922 to 96597.83) | 54.948(39.706 to 73.493) | 66885.999(47130.06 to 91541.737) | 52.088(36.703 to 71.288) | -4.691  (-4.884 to -4.497) | -0.125(-0.192 to -0.048) |
| 2 to 4 | 290173.63(210797.349 to 368655.352) | 78.943(57.349 to 100.295) | 69033.727(46757.325 to 97738.94) | 16.769(11.358 to 23.742) | 64670.734(42280.124 to 92970.861) | 15.802(10.331 to 22.718) | 59209.45(37892.313 to 86988.189) | 14.69(9.401 to 21.582) | -5.066  (-5.358 to -4.773) | -0.124 (-0.194 to -0.052) |
| 5 to 9 | 70288.54(40219.911 to 99719.459) | 12.045(6.893 to 17.089) | 24787.54(14177.626 to 41362.336) | 3.67(2.099 to 6.125) | 22647.194(12583.74 to 38220.064) | 3.326(1.848 to 5.613) | 20943.363(11638.436 to 34889.208) | 3.048(1.694 to 5.078) | -3.559  (-3.827 to -3.29) | -0.169 (-0.23 to -0.114) |
| 10 to 14 | 33013.443(20546.441 to 50102.056) | 6.163(3.836 to 9.353) | 14239.001(8401.941 to 23784.09) | 2.182(1.288 to 3.645) | 13576.566(8080.453 to 22446.815) | 2.055(1.223 to 3.398) | 12873.035(7498.045 to 21217.572) | 1.931(1.125 to 3.183) | -3.221  (-3.394 to -3.048) | -0.115(-0.171 to -0.059) |
| High SDI | | | | | | | | | | |
| 0 to 14 | 2207.306(1723.336 to 2935.859) | 1.188(0.927 to 1.58) | 571.461(518.62 to 632.697) | 0.328(0.298 to 0.363) | 504.365(448.591 to 563.29) | 0.291(0.259 to 0.325) | 474.56(410.766 to 539.035) | 0.275(0.238 to 0.312) | -4.243  (-4.5 to -3.985) | -0.162(-0.226 to -0.1) |
| Neonatal | 241.128(179.448 to 351.74) | 25.365(18.877 to 37.001) | 93.609(85.868 to 104.038) | 11.605(10.645 to 12.898) | 84.683(75.571 to 95.81) | 10.725(9.571 to 12.134) | 79.664(68.988 to 91.49) | 10.163(8.801 to 11.672) | -2.647  (-2.876 to -2.416) | -0.124 (-0.21 to -0.04) |
| 1 to 5 months | 771.294(593.263 to 1073.841) | 14.704(11.31 to 20.472) | 199.003(182.195 to 223.105) | 4.429(4.055 to 4.965) | 183.899(162.477 to 206.609) | 4.186(3.698 to 4.703) | 172.1(146.314 to 198.16) | 3.959(3.366 to 4.558) | -4.641  (-4.827 to -4.455) | -0.106(-0.201 to -0.017) |
| 6 to 11 months | 539.841(355.533 to 810.142) | 8.789(5.788 to 13.19) | 89.694(76.955 to 106.078) | 1.678(1.44 to 1.985) | 71.047(58.693 to 85.675) | 1.362(1.126 to 1.643) | 65.897(53.284 to 79.988) | 1.285(1.039 to 1.56) | -5.294  (-5.694 to -4.893) | -0.234 (-0.313 to -0.147) |
| 12 to 23 months | 385.967(276.538 to 567.933) | 3.128(2.241 to 4.602) | 103.399(89.451 to 121.165) | 0.944(0.816 to 1.106) | 89.576(77.058 to 104.773) | 0.839(0.721 to 0.981) | 84.274(71.539 to 100.033) | 0.808(0.686 to 0.959) | -4.029  (-4.306 to -3.752) | -0.144 (-0.213 to -0.071) |
| 2 to 4 | 151.207(98.178 to 230.715) | 0.408(0.265 to 0.623) | 32.128(26.541 to 39.619) | 0.094(0.077 to 0.115) | 25.174(20.958 to 31.716) | 0.074(0.062 to 0.094) | 22.697(18.362 to 28.72) | 0.068(0.055 to 0.087) | -5.405  (-5.624 to -5.185) | -0.268(-0.338 to -0.193) |
| 5 to 9 | 77.121(48.739 to 117.038) | 0.123(0.078 to 0.187) | 31.942(22.523 to 53.535) | 0.054(0.038 to 0.091) | 28.694(19.811 to 48.937) | 0.049(0.034 to 0.083) | 28.844(19.974 to 48.342) | 0.049(0.034 to 0.082) | -2.296  (-2.622 to -1.968) | -0.098(-0.136 to -0.045) |
| 10 to 14 | 40.748(26.852 to 59.201) | 0.066(0.044 to 0.096) | 21.686(15.997 to 33.416) | 0.036(0.027 to 0.056) | 21.291(15.502 to 32.649) | 0.036(0.026 to 0.055) | 21.085(15.261 to 32.606) | 0.035(0.025 to 0.054) | -1.488  (-1.82 to -1.155) | -0.036(-0.072 to 0.008) |
| High-middle SDI | | | | | | | | | | |
| 0 to 14 | 34407.118(27556.474 to 41286.667) | 12.575(10.071 to 15.089) | 2252.636(1805.589 to 2734.491) | 0.974(0.78 to 1.182) | 2008.344(1601.775 to 2441.166) | 0.866(0.691 to 1.053) | 1872.697(1455.73 to 2294.971) | 0.811(0.63 to 0.994) | -8.757  (-8.925 to -8.588) | -0.167(-0.236 to -0.088) |
| Neonatal | 2702.65 (2019.147 to 3407.945) | 195.093(145.753 to 246.005) | 272.504(210.955 to 369.207) | 26.909(20.831 to 36.458) | 244.803(186.259 to 339.573) | 26.071(19.836 to 36.164) | 231.295(172.811 to 323.417) | 25.942(19.382 to 36.275) | -7.564  (-7.755 to -7.372) | -0.036 (-0.126 to 0.059) |
| 1 to 5 months | 9477.293(7360.078 to 11811.099) | 123.874(96.201 to 154.379) | 701.233 (541.088 to 875.484) | 12.301(9.492 to 15.357) | 654.872(510.656 to 824.146) | 12.366(9.643 to 15.563) | 611.614(470.458 to 774.957) | 12.238(9.414 to 15.506) | -9.499  (-9.813 to -9.184) | -0.005(-0.105 to 0.1) |
| 6 to 11 months | 8905.547(6977.435 to 11015.014) | 98.928(77.51 to 122.362) | 390.52(296.237 to 501.66) | 5.648(4.285 to 7.256) | 340.567(254.152 to 441.263) | 5.286(3.945 to 6.849) | 315.158(228.898 to 420.906) | 5.24(3.806 to 6.998) | -10.346  (-10.588 to -10.103) | -0.072(-0.18 to 0.056) |
| 12 to 23 months | 8031.189(5986.054 to 10354.998) | 43.743(32.604 to 56.4) | 439.696(348.708 to 550.591) | 2.983(2.366 to 3.735) | 383.474(298.985 to 484.743) | 2.78(2.168 to 3.515) | 355.288(270.283 to 450.443) | 2.773(2.109 to 3.515) | -9.3  (-9.533 to -9.066) | -0.07(-0.169 to 0.042) |
| 2 to 4 | 3968.765(2845.288 to 5190.029) | 7.024(5.035 to 9.185) | 221.428(165.427 to 294.17) | 0.459(0.343 to 0.609) | 181.376(134.337 to 243.066) | 0.382(0.283 to 0.512) | 164.774(117.199 to 220.148) | 0.364(0.259 to 0.486) | -9.025  (-9.232 to -8.818) | -0.208 (-0.299 to -0.107) |
| 5 to 9 | 874.47(517.464 to 1294.005) | 0.96(0.568 to 1.421) | 132.076(76.263 to 235.573) | 0.168(0.097 to 0.299) | 115.314(65.695 to 208.945) | 0.143(0.082 to 0.26) | 109.479(63.275 to 201.963) | 0.133(0.077 to 0.245) | -5.991  (-6.173 to -5.809) | -0.207 (-0.289 to -0.118) |
| 10 to 14 | 447.203(267.335 to 700.464) | 0.499(0.298 to 0.781) | 95.178(54.829 to 156.369) | 0.125(0.072 to 0.206) | 87.938(49.861 to 148.201) | 0.114(0.064 to 0.191) | 85.089(47.23 to 141.089) | 0.108(0.06 to 0.18) | -5.044  (-5.265 to -4.823) | -0.135(-0.205 to -0.062) |
| Middle SDI | | | | | | | | | | |
| 0 to 14 | 304638.576(238427.702 to 360187.622) | 52.777(41.307 to 62.401) | 35309.018(27713.176 to 45251.135) | 6.199(4.866 to 7.945) | 30255.321(23460.748 to 38801.408) | 5.314(4.12 to 6.815) | 28625.018(21973.608 to 37113.689) | 5.05(3.876 to 6.547) | -6.965  (-7.137 to -6.793) | -0.185(-0.246 to -0.119) |
| Neonatal | 28962.106(19014.956 to 38580.227) | 919.853(603.926 to 1225.33) | 2525.815(1890.152 to 3347.784) | 95.858(71.734 to 127.053) | 2222.047(1665.089 to 2962.777) | 88.577(66.375 to 118.104) | 2127.097(1603.747 to 2839.348) | 87.923(66.29 to 117.363) | -7.378  (-7.474 to -7.281) | -0.083(-0.153 to -0.009) |
| 1 to 5 months | 96306.668(74696.247 to 111905.314) | 559.598(434.029 to 650.235) | 10898.522(8381.991 to 13864.615) | 74.177(57.049 to 94.364) | 9228.204(7090.045 to 11850.97) | 65.886(50.62 to 84.612) | 8895.907(6971.872 to 11577.086) | 66.09(51.796 to 86.009) | -7.389  (-7.514 to -7.263) | -0.109 (-0.176 to -0.038) |
| 6 to 11 months | 70680.735(56915.359 to 83681.448) | 353.881(284.961 to 418.972) | 8701.78(6538.772 to 11420.922) | 49.534(37.221 to 65.013) | 7573.218(5631.229 to 9949.576) | 45.131(33.558 to 59.292) | 7088.08(5251.859 to 9450.162) | 44.235(32.776 to 58.976) | -6.889  (-7.051 to -6.727) | -0.107(-0.191 to -0.02) |
| 12 to 23 months | 55108.146(42433.947 to 66884.771) | 137.789(106.099 to 167.234) | 6184.845(4642.88 to 8140.697) | 16.904(12.69 to 22.25) | 4811.275(3577.781 to 6441.313) | 13.719(10.202 to 18.367) | 4531.403(3350.865 to 6105.677) | 13.557(10.025 to 18.267) | -6.783  (-7.095 to -6.469) | -0.198(-0.283 to -0.113) |
| 2 to 4 | 37424.411(25979.308 to 46823.046) | 31.133(21.612 to 38.951) | 3395.342(2510.276 to 4684.786) | 2.928(2.165 to 4.04) | 3016.566(2195.773 to 4222.869) | 2.631(1.915 to 3.683) | 2796.915(2027.752 to 3906.865) | 2.513(1.822 to 3.511) | -7.689  (-7.837 to -7.541) | -0.142(-0.234 to -0.048) |
| 5 to 9 | 10574.823(6174.275 to 15570.507) | 5.465(3.191 to 8.047) | 2129.728(1267.478 to 3558.96) | 1.108(0.659 to 1.851) | 2029.547(1131.539 to 3422.522) | 1.044(0.582 to 1.761) | 1890.193(1075.625 to 3152.693) | 0.959(0.546 to 1.6) | -5.152  (-5.283 to -5.02) | -0.134 (-0.216 to -0.035) |
| 10 to 14 | 5581.687(3284.693 to 8585.919) | 3.047(1.793 to 4.687) | 1472.988(847.985 to 2408.508) | 0.776(0.447 to 1.269) | 1374.465 (800.303 to 2303.179) | 0.716(0.417 to 1.2) | 1295.425(757.141 to 2093.112) | 0.671(0.392 to 1.084) | -4.565  (-4.726 to -4.404) | -0.136(-0.201 to -0.065) |
| Low-middle SDI | | | | | | | | | | |
| 0 to 14 | 794757.451(647653.807 to 931120.672) | 168.341(137.182 to 197.224) | 125117.12(98855.515 to 161875.123) | 21.506(16.992 to 27.824) | 105945.284(82882.599 to 137738.172) | 18.235(14.265 to 23.707) | 97622.489(76594.921 to 126806.086) | 16.836(13.21 to 21.869) | -6.467  (-6.653 to -6.281) | -0.217 (-0.28 to -0.156) |
| Neonatal | 94516.948(67570.016 to 120342.59) | 3264.715(2333.94 to 4156.76) | 9704.836(7301.79 to 13966.145) | 322.33(242.517 to 463.862) | 8617.253(6328.245 to 12588.987) | 290.596(213.405 to 424.533) | 8030.619(5911.271 to 11746.559) | 273.745(201.501 to 400.413) | -7.392  (-7.507 to -7.277) | -0.151(-0.234 to -0.08) |
| 1 to 5 months | 248093.724(200528.166 to 288428.277) | 1588.486(1283.935 to 1846.738) | 38611.16(29501.781 to 52963.942) | 233.203(178.184 to 319.891) | 30407.237(22080.132 to 42207.782) | 186.32(135.295 to 258.627) | 28429.934(20752.169 to 39843.985) | 176.164(128.59 to 246.891) | -6.231  (-6.409 to -6.053) | -0.245(-0.317 to -0.168) |
| 6 to 11 months | 147847.73(117332.426 to 180557.96) | 822.866(653.029 to 1004.919) | 30461.185(22280.252 to 40438.741) | 156.976(114.817 to 208.393) | 25710.698(18365.808 to 34820.846) | 134.281(95.92 to 181.861) | 23533.085(16987.961 to 32539.31) | 124.436(89.827 to 172.058) | -5.024  (-5.226 to -4.821) | -0.207 (-0.293 to -0.12) |
| 12 to 23 months | 126147.138(95321.589 to 153729.048) | 360.311(272.264 to 439.092) | 18370.973(13892.704 to 23969.594) | 47.1(35.618 to 61.454) | 16113.045(12059.484 to 21478.345) | 41.837(31.312 to 55.768) | 14781.824(10993.276 to 19938.8) | 38.864(28.903 to 52.423) | -6.459  (-6.571 to -6.346) | -0.175(-0.26 to -0.089) |
| 2 to 4 | 122725.01(92575.741 to 156238.982) | 120.329(90.768 to 153.188) | 13196.158(9666.599 to 18030.985) | 11.222(8.221 to 15.334) | 11885.684(8551.369 to 16832.639) | 10.189(7.331 to 14.43) | 10726.685(7713.808 to 15228.381) | 9.282(6.675 to 13.178) | -7.678  (-7.943 to -7.412) | -0.173 (-0.26 to -0.085) |
| 5 to 9 | 37612.32(22658.703 to 53149.875) | 23.492(14.152 to 33.196) | 9019.47(5158.278 to 15032.287) | 4.618(2.641 to 7.697) | 7798.786(4300.865 to 13058.971) | 3.995(2.203 to 6.689) | 7036.474(3970.467 to 11456.114) | 3.611(2.038 to 5.879) | -5.307  (-5.607 to -5.006) | -0.218 (-0.287 to -0.139) |
| 10 to 14 | 17814.581(11258.051 to 27640.162) | 12.861(8.127 to 19.954) | 5753.339(3408.988 to 9691.707) | 3.014(1.786 to 5.076) | 5412.582(3259.987 to 9007.352) | 2.816(1.696 to 4.687) | 5083.869(2996.506 to 8608.888) | 2.629(1.549 to 4.451) | -4.96  (-5.177 to -4.743) | -0.128(-0.192 to -0.041) |
| Low SDI | | | | | | | | | | |
| 0 to 14 | 602562.58(450215.548 to 752457.754) | 263.227(196.675 to 328.708) | 285213.157(213939.302 to 377664.937) | 63.714(47.792 to 84.367) | 261607.399(189381.236 to 352694.394) | 57.617(41.71 to 77.678) | 245328.66(176547.497 to 335720.04) | 53.306(38.361 to 72.947) | -4.919  (-5.173 to -4.665) | -0.163(-0.218 to -0.108) |
| Neonatal | 47021.847(32072.679 to 61453.07) | 2846.514(1941.552 to 3720.123) | 15890.989(12078.969 to 21840.059) | 597.577(454.227 to 821.291) | 14603.572(10860.812 to 20344.714) | 545.058 (405.365 to 759.338) | 13981.85(10301.986 to 19666.869) | 516.121(380.283 to 725.975) | -5.245  (-5.502 to -4.987) | -0.136(-0.191 to -0.085) |
| 1 to 5 months | 164136.1(125578.865 to 195296.719) | 1859.456(1422.651 to 2212.467) | 74846.878(58424.753 to 95088.098) | 516.376(403.078 to 656.022) | 65967.135(50260.075 to 84822.245) | 451.343(343.876 to 580.348) | 62977.057(47694.514 to 81508.535) | 426.377(322.909 to 551.842) | -4.552  (-4.817 to -4.287) | -0.174(-0.225 to -0.124) |
| 6 to 11 months | 115131.358(83151.608 to 145834.815) | 1154.058(833.498 to 1461.824) | 67162.292(47636.158 to 94163.706) | 401.01(284.424 to 562.229) | 61423.045(41814.178 to 87538.97) | 363.264(247.295 to 517.717) | 57595.031(39107.936 to 83588.862) | 337.555(229.205 to 489.901) | -3.385  (-3.565 to -3.206) | -0.158(-0.223 to -0.088) |
| 12 to 23 months | 120253.659(85124.628 to 156218.248) | 639.315(452.555 to 830.517) | 54806.782(39077.367 to 75865.785) | 166.517(118.727 to 230.499) | 50747.108(35407.768 to 71009.378) | 152.489(106.396 to 213.374) | 47058.416(32201.506 to 66792.655) | 140.022(95.815 to 198.741) | -4.8  (-5.01 to -4.589) | -0.159(-0.227 to -0.079) |
| 2 to 4 | 125772.791(82805.405 to 170225.175) | 244.074(160.692 to 330.338) | 52152.883(33404.511 to 76375.54) | 54.783(35.089 to 80.227) | 49527.992(30913.557 to 73982.543) | 51.433(32.103 to 76.828) | 45466.248(27364.43 to 69997.377) | 46.668(28.087 to 71.847) | -5.352  (-5.581 to -5.122) | -0.148(-0.222 to -0.076) |
| 5 to 9 | 21128.615(10617.637 to 32331.855) | 27.896(14.018 to 42.687) | 13463.388(7508.536 to 22472.508) | 8.988(5.013 to 15.002) | 12664.001(6952.799 to 21418.967) | 8.349(4.584 to 14.12) | 11868.028(6443.283 to 20267.811) | 7.732(4.198 to 13.204) | -3.528  (-3.734 to -3.321) | -0.14(-0.209 to -0.082) |
| 10 to 14 | 9118.208(5080.598 to 13770.161) | 14.618(8.145 to 22.076) | 6889.945(4038.338 to 11744.462) | 5.072(2.973 to 8.646) | 6674.547(3925.417 to 11103.15) | 4.816(2.833 to 8.012) | 6382.03(3630.916 to 10703.466) | 4.521(2.572 to 7.583) | -3.279  (-3.515 to -3.042) | -0.109(-0.172 to -0.051) |
| World Bank High Income | | | | | | | | | | |
| 0 to 14 | 3021.885(2519.652 to 3806.414) | 1.428(1.191 to 1.799) | 858.393 (786.395 to 940.043) | 0.438(0.402 to 0.48) | 755.397(671.533 to 835.814) | 0.388(0.345 to 0.429) | 709.138 (609.328 to 803.443) | 0.366(0.315 to 0.415) | -3.947  (-4.264 to -3.629) | -0.164(-0.23 to -0.098) |
| Neonatal | 308.327(242.981 to 423.951) | 28.878(22.758 to 39.707) | 127.429(118.183 to 139.14) | 14.138(13.112 to 15.438) | 115.252(103.465 to 127.904) | 13.012(11.682 to 14.441) | 108.219(93.839 to 124.033) | 12.288(10.655 to 14.083) | -2.431  (-2.791 to -2.07) | -0.131(-0.215 to -0.048) |
| 1 to 5 months | 1089.888(901.307 to 1394.501) | 18.503(15.301 to 23.674) | 293.303 (272.065 to 319.153) | 5.846(5.422 to 6.361) | 267.972(239.181 to 295.806) | 5.442(4.857 to 6.007) | 250.548(212.588 to 286.547) | 5.131(4.354 to 5.869) | -4.549  (-4.792 to -4.306) | -0.122 (-0.214 to -0.039) |
| 6 to 11 months | 795.743(592.194 to 1076.98) | 11.539(8.587 to 15.617) | 143.99(124.627 to 166.498) | 2.415(2.091 to 2.793) | 115.138(95.519 to 135.798) | 1.973(1.637 to 2.327) | 107.838(86.391 to 128.709) | 1.873(1.501 to 2.236) | -4.951  (-5.463 to -4.436) | -0.224 (-0.302 to -0.145) |
| 12 to 23 months | 510.53(396.6 to 685.789) | 3.686(2.864 to 4.952) | 176.838(154.484 to 201.51) | 1.451(1.267 to 1.653) | 154.362(133.5 to 176.402) | 1.295(1.12 to 1.48) | 144.913(121.293 to 170.93) | 1.241(1.039 to 1.464) | -3.141  (-3.455 to -2.826) | -0.144(-0.222 to -0.072) |
| 2 to 4 | 179.075(124.731 to 259.387) | 0.432(0.301 to 0.626) | 47.336(40.784 to 55.555) | 0.124(0.107 to 0.146) | 38.849(33.568 to 45.68) | 0.103(0.089 to 0.122) | 33.611(28.274 to 40.083) | 0.091(0.077 to 0.109) | -4.658  (-4.975 to -4.339) | -0.264 (-0.326 to -0.202) |
| 5 to 9 | 90.485(63.962 to 126.154) | 0.128(0.09 to 0.178) | 41.544(31.904 to 62.823) | 0.063(0.048 to 0.095) | 37.227(28.098 to 56.408) | 0.057(0.043 to 0.086) | 37.385(28.036 to 55.892) | 0.057(0.043 to 0.085) | -2.004  (-2.331 to -1.677) | -0.092(-0.126 to -0.049) |
| 10 to 14 | 47.836(35.329 to 64.56) | 0.067(0.049 to 0.09) | 27.951(22.05 to 39.605) | 0.041(0.033 to 0.059) | 26.597(20.593 to 37.891) | 0.039(0.03 to 0.056) | 26.624(20.667 to 37.96) | 0.039(0.03 to 0.056) | -1.072  (-1.403 to -0.739) | -0.053(-0.089 to -0.015) |
| World Bank Upper Middle Income | | | | | | | | | | |
| 0 to 14 | 192157.329(167831.287 to 217000.232) | 31.473(27.489 to 35.542) | 15783.45(13161.534 to 18762.91) | 2.945(2.455 to 3.5) | 13542.46(11162.812 to 16370.423) | 2.521(2.078 to 3.048) | 12682.427(10371.818 to 15327.998) | 2.37(1.938 to 2.864) | -7.798  (-7.999 to -7.597) | -0.195(-0.247 to -0.144) |
| Neonatal | 11503.361(9940.947 to 13208.44) | 351.633(303.873 to 403.753) | 775.43(624.699 to 969.241) | 32.007(25.785 to 40.007) | 681.443(539.558 to 856.077) | 30.325(24.011 to 38.097) | 639.161(500.911 to 800.365) | 29.945(23.468 to 37.497) | -8.427  (-8.674 to -8.179) | -0.064(-0.122 to -0.01) |
| 1 to 5 months | 64832.128(56881.618 to 73169.332) | 361.243(316.943 to 407.698) | 4837.469(3960.262 to 5763.935) | 35.543(29.098 to 42.351) | 4361.956(3574.293 to 5208.528) | 34.475(28.25 to 41.166) | 4116.976(3379.903 to 4974.718) | 34.47(28.299 to 41.652) | -8.486  (-8.71 to -8.26) | -0.03(-0.091 to 0.033) |
| 6 to 11 months | 49069.299(41728.487 to 56997.31) | 234.678(199.57 to 272.594) | 3903.451(3184.463 to 4811.618) | 23.706(19.34 to 29.221) | 3530.517(2808.442 to 4389.028) | 22.976(18.277 to 28.563) | 3286.986(2607.653 to 4077.858) | 22.912(18.177 to 28.425) | -8.374  (-8.652 to -8.096) | -0.033 (-0.111 to 0.044) |
| 12 to 23 months | 41537.891(34605.02 to 48899.865) | 98.656(82.19 to 116.142) | 3668.814(2979.648 to 4469.889) | 10.501(8.529 to 12.794) | 2618.905(2070.525 to 3207.762) | 7.991(6.318 to 9.788) | 2423.159(1890.502 to 2995.53) | 7.947(6.2 to 9.824) | -7.415  (-7.821 to -7.007) | -0.243 (-0.315 to -0.167) |
| 2 to 4 | 17954.556(14660.732 to 22008.923) | 14.113(11.524 to 17.3) | 1227.511(957.147 to 1539.952) | 1.088(0.848 to 1.365) | 1059.963(798.746 to 1392.657) | 0.951(0.716 to 1.249) | 966.315(722.11 to 1275.025) | 0.904(0.675 to 1.192) | -8.55  (-8.896 to -8.202) | -0.169 (-0.274 to -0.082) |
| 5 to 9 | 5013.235(3469.652 to 6536.193) | 2.474(1.712 to 3.226) | 867.847(632.936 to 1258.826) | 0.48(0.35 to 0.696) | 794.379(566.38 to 1160.111) | 0.43(0.307 to 0.628) | 764.946(538.335 to 1138.796) | 0.402(0.283 to 0.599) | -5.484  (-5.705 to -5.263) | -0.162(-0.214 to -0.111) |
| 10 to 14 | 2246.859(1592.848 to 3164.598) | 1.144(0.811 to 1.611) | 502.928(350.75 to 707.016) | 0.287(0.2 to 0.404) | 495.297(342.281 to 701.558) | 0.279(0.193 to 0.395) | 484.884(332.141 to 697.574) | 0.27(0.185 to 0.389) | -4.499  (-4.813 to -4.183) | -0.059(-0.099 to -0.021) |
| World Bank Lower Middle Income | | | | | | | | | | |
| 0 to 14 | 1203982.15(946751.783 to 1417065.41) | 157.796(124.083 to 185.723) | 284748.384(223042.59 to 360166.562) | 29.106(22.798 to 36.815) | 247622.66(191844.926 to 323520.598) | 25.295(19.598 to 33.049) | 230353.301(176087.552 to 302402.045) | 23.548(18.001 to 30.913) | -5.175  (-5.345 to -5.004) | -0.191(-0.25 to -0.12) |
| Neonatal | 138187.331(93222.422 to 173573.632) | 3029.167(2043.503 to 3804.86) | 18946.457(14600.362 to 25279.07) | 377.499(290.905 to 503.673) | 16798.97(12772.657 to 23043.856) | 339.122(257.843 to 465.188) | 15791.51(11922.213 to 21904.57) | 321.865(243.001 to 446.463) | -6.515  (-6.627 to -6.404) | -0.147 (-0.226 to -0.084) |
| 1 to 5 months | 353117.519(282723.017 to 411726.004) | 1430.762(1145.537 to 1668.232) | 80549.771(64188.714 to 104612.264) | 291.92(232.626 to 379.124) | 65010.699(51123.65 to 83711.246) | 238.539(187.585 to 307.156) | 61470.221(48220.318 to 80349.527) | 227.824(178.716 to 297.795) | -5.165  (-5.302 to -5.029) | -0.22(-0.285 to -0.15) |
| 6 to 11 months | 222974.087(175734.976 to 269070.47) | 783.473 (617.487 to 945.443) | 68210.893(52449.134 to 88982.793) | 211.055(162.286 to 275.327) | 58791.888(43748.377 to 78988.021) | 184.009(136.925 to 247.22) | 54714.092(40645.265 to 75149.814) | 173.17(128.642 to 237.849) | -3.908  (-4.026 to -3.791) | -0.18(-0.257 to -0.094) |
| 12 to 23 months | 202558.225(148857.046 to 249763.991) | 363.027(266.783 to 447.63) | 49620.038(36411.745 to 64190.516) | 76.321(56.005 to 98.732) | 44486.4(32008.473 to 59503.809) | 69.155(49.758 to 92.5) | 41212.706(29331.495 to 56256.119) | 64.801(46.119 to 88.454) | -4.822  (-4.949 to -4.695) | -0.151(-0.231 to -0.052) |
| 2 to 4 | 203329.282(147603.526 to 256526.823) | 123.88(89.929 to 156.291) | 41063.566(27843.108 to 56528.284) | 20.845(14.134 to 28.696) | 38604.127(25056.785 to 54486.441) | 19.735(12.809 to 27.854) | 35168.364(22708.096 to 51397.717) | 18.136(11.711 to 26.506) | -5.535  (-5.753 to -5.316) | -0.13(-0.21 to -0.039) |
| 5 to 9 | 56853.337(32269.066 to 81399.865) | 21.872(12.414 to 31.316) | 16294.293(9484.314 to 27655.941) | 4.934(2.872 to 8.375) | 14459.229(8309.463 to 24739.252) | 4.375(2.514 to 7.485) | 13134.593(7495.747 to 22084.928) | 3.979(2.271 to 6.691) | -4.69  (-4.97 to -4.409) | -0.194 (-0.264 to -0.121) |
| 10 to 14 | 26962.369(16985.121 to 42077.571) | 11.96(7.534 to 18.665) | 10063.366(6003.509 to 16885.988) | 3.133(1.869 to 5.258) | 9471.347(5673.294 to 15860.415) | 2.92(1.749 to 4.891) | 8861.815(5150.236 to 15064.649) | 2.709(1.574 to 4.605) | -4.355  (-4.556 to -4.153) | -0.135(-0.201 to -0.064) |
| World Bank Low Income | | | | | | | | | | |
| 0 to 14 | 339411.458(250943.227 to 432800.253) | 222.593(164.574 to 283.839) | 147073.132(106728.624 to 205322.536) | 49.986(36.274 to 69.783) | 138400.043(99006.934 to 197549.797) | 46.306(33.126 to 66.096) | 130178.404(91870.18 to 186314.75) | 42.901(30.276 to 61.401) | -5.539  (-5.828 to -5.25) | -0.142(-0.198 to -0.093) |
| Neonatal | 23445.652(17034.877 to 30354.257) | 2073.974(1506.884 to 2685.101) | 8638.436(6085.199 to 12708.686) | 484.919(341.593 to 713.403) | 8176.687(5642.697 to 12096.04) | 455.44(314.297 to 673.747) | 7911.629(5399.649 to 11703.202) | 435.687(297.354 to 644.485) | -5.264  (-5.598 to -4.929) | -0.102(-0.156 to -0.046) |
| 1 to 5 months | 99745.506(72824.087 to 121189.878) | 1653.163(1206.972 to 2008.577) | 39576.248(29793.491 to 52331.504) | 407.258(306.589 to 538.516) | 36800.69(27315.924 to 48749.384) | 375.484(278.709 to 497.399) | 35248.837(26226.816 to 47209.833) | 355.834(264.757 to 476.579) | -5.389  (-5.745 to -5.032) | -0.126 (-0.179 to -0.072) |
| 6 to 11 months | 70266.049(49296.614 to 91606.202) | 1034.03(725.446 to 1348.07) | 34547.131(23058.435 to 51432.465) | 307.545(205.271 to 457.862) | 32680.994(21198.545 to 49208.005) | 288.148(186.907 to 433.867) | 30488.297(19518.674 to 46608.102) | 266.426(170.566 to 407.291) | -4.179  (-4.428 to -3.93) | -0.134 (-0.204 to -0.064) |
| 12 to 23 months | 65319.357(46153.495 to 86853.85) | 511.704(361.561 to 680.403) | 26439.988(18169.018 to 38433.049) | 119.843(82.354 to 174.204) | 24884.77(16656.56 to 36789.911) | 111.494(74.629 to 164.835) | 23030.386(14800.06 to 34645.341) | 102.159(65.65 to 153.681) | -5.515  (-5.768 to -5.262) | -0.148(-0.217 to -0.079) |
| 2 to 4 | 68579.256(44492.703 to 95139.77) | 199.099(129.171 to 276.21) | 26659.524(16897.423 to 40891.497) | 42.051(26.653 to 64.5) | 24933.83(15326.581 to 39180.232) | 38.797(23.848 to 60.964) | 23009.006(13817.255 to 36813.524) | 35.335(21.219 to 56.535) | -6.02  (-6.218 to -5.821) | -0.16(-0.234 to -0.085) |
| 5 to 9 | 8310.283(4011.521 to 14023.834) | 16.721(8.072 to 28.217) | 7572.917(3988.643 to 12222.73) | 7.743(4.078 to 12.497) | 7345.497(3776.982 to 12272.055) | 7.392(3.801 to 12.349) | 6996.084(3612.984 to 11841.813) | 6.932(3.58 to 11.734) | -2.486  (-2.644 to -2.327) | -0.105(-0.187 to -0.04) |
| 10 to 14 | 3745.354(1892.572 to 6189.868) | 9.001(4.548 to 14.875) | 3638.889(2050.36 to 6080.167) | 4.124(2.324 to 6.891) | 3577.574(1970.665 to 5851.031) | 3.976(2.19 to 6.502) | 3494.166(1925.225 to 5871.288) | 3.811(2.1 to 6.403) | -2.595  (-2.758 to -2.432) | -0.076(-0.133 to -0.021) |
| North Africa and Middle East | | | | | | | | | | |
| 0 to 14 | 91953.216(69091.57 to 117564.012) | 65.454(49.181 to 83.684) | 15431.41(11227.372 to 22585.974) | 8.468(6.161 to 12.393) | 13127.528(9400.637 to 19643.202) | 7.174(5.137 to 10.734) | 12021.149(8527.802 to 18090.537) | 6.557(4.652 to 9.868) | -6.614  (-6.898 to -6.33) | -0.226 (-0.289 to -0.158) |
| Neonatal | 12388.491(8802.387 to 17390.484) | 1505.227(1069.508 to 2112.98) | 1746.354(1156.542 to 3044.189) | 183.601(121.592 to 320.048) | 1516.185(970.479 to 2681.657) | 163.968(104.952 to 290.008) | 1418.454(914.455 to 2493.968) | 156.913(101.159 to 275.889) | -7.253  (-7.611 to -6.894) | -0.145(-0.233 to -0.068) |
| 1 to 5 months | 30687.043(22922.826 to 40076.397) | 685.051(511.724 to 894.657) | 4852.594(3531.15 to 6982.775) | 92.064(66.994 to 132.478) | 4071.246(2867.414 to 5957.008) | 79.414(55.932 to 116.199) | 3772.479(2624.281 to 5690.202) | 75.355(52.42 to 113.662) | -7.871  (-8.245 to -7.495) | -0.181(-0.277 to -0.077) |
| 6 to 11 months | 22782.66(16837.57 to 29347.916) | 438.693(324.217 to 565.11) | 4341.95(2903.628 to 6334.783) | 69.611(46.552 to 101.561) | 3669.261(2331.33 to 5630.717) | 60.459(38.414 to 92.778) | 3283.211(2062.409 to 5085.562) | 55.518(34.875 to 85.995) | -5.165  (-5.493 to -4.835) | -0.202(-0.313 to -0.082) |
| 12 to 23 months | 15007.431(10604.47 to 19987.011) | 145.993(103.161 to 194.435) | 2607.156(1778.736 to 3904.796) | 20.609(14.061 to 30.867) | 2248.048(1541.097 to 3397.918) | 18.223(12.493 to 27.544) | 2045.113(1383.289 to 3100.041) | 17.031(11.519 to 25.816) | -6.347  (-6.613 to -6.081) | -0.174 (-0.269 to -0.065) |
| 2 to 4 | 9518.935(6419.27 to 13532.214) | 31.257(21.078 to 44.435) | 1258.172 (805.363 to 2064.943) | 3.26(2.087 to 5.35) | 1056.505(648.226 to 1774.712) | 2.777(1.704 to 4.665) | 954.227(584.476 to 1563.219) | 2.558(1.567 to 4.19) | -7.708  (-8.219 to -7.194) | -0.215(-0.313 to -0.124) |
| 5 to 9 | 1076.556(424.976 to 1744.373) | 2.267(0.895 to 3.673) | 408.243 (212.254 to 693.422) | 0.657(0.342 to 1.117) | 363.396(174.493 to 639.176) | 0.578(0.278 to 1.017) | 347.958(169.718 to 615.3) | 0.551(0.269 to 0.974) | -4.081  (-4.194 to -3.968) | -0.163 (-0.28 to -0.076) |
| 10 to 14 | 492.101(241.495 to 783.344) | 1.178(0.578 to 1.876) | 216.941(106.582 to 375.93) | 0.384(0.189 to 0.666) | 202.888(93.565 to 360.86) | 0.352(0.162 to 0.626) | 199.709(92.608 to 352.523) | 0.339(0.157 to 0.598) | -3.854  (-3.939 to -3.77) | -0.119(-0.201 to -0.055) |
| South Asia | | | | | | | | | | |
| 0 to 14 | 679060.009(551103.007 to 817209.497) | 156.696(127.169 to 188.574) | 93626.437(65839.588 to 130178.792) | 18.192(12.793 to 25.295) | 77620.341(52694.331 to 108080.89) | 15.187(10.31 to 21.147) | 69139.936(46159.464 to 95752.928) | 13.636(9.104 to 18.885) | -6.82  (-6.995 to -6.645) | -0.25(-0.336 to -0.144) |
| Neonatal | 89771.771(63463.452 to 114129.993) | 3501.999(2475.711 to 4452.214) | 7875.157(5188.797 to 12018.1) | 320.956(211.472 to 489.803) | 7205.139(4552.809 to 11054.015) | 298.696(188.741 to 458.255) | 6505.762(4065.343 to 9947.821) | 273.52(170.918 to 418.234) | -7.687  (-7.841 to -7.533) | -0.148(-0.268 to -0.043) |
| 1 to 5 months | 203510.287(163983.085 to 241206.637) | 1470.963(1185.262 to 1743.43) | 28733.197(17258.308 to 42972.058) | 212.941(127.901 to 318.465) | 20915.675(12253.534 to 30731.217) | 157.52(92.284 to 231.443) | 18789.772(10897.874 to 28225.935) | 143.517(83.238 to 215.59) | -6.132  (-6.298 to -5.966) | -0.326 (-0.434 to -0.2) |
| 6 to 11 months | 106754.935(80660.648 to 134787.492) | 668.939(505.429 to 844.595) | 20064.949(12106.075 to 29574.072) | 126.759(76.479 to 186.832) | 16249.52(9173.605 to 24067.005) | 104.186(58.818 to 154.309) | 14498.031(8121.055 to 22050.099) | 94.324(52.836 to 143.458) | -4.978  (-5.147 to -4.808) | -0.256 (-0.388 to -0.092) |
| 12 to 23 months | 101994.385(74733.248 to 130384.798) | 325.4(238.427 to 415.976) | 11152.258(7134.925 to 15817.886) | 34.737(22.224 to 49.27) | 10148.184(6318.622 to 14724.242) | 32.074(19.971 to 46.537) | 9022.93(5568.971 to 12731.633) | 28.949(17.867 to 40.847) | -7.314  (-7.433 to -7.194) | -0.167(-0.3 to 0.006) |
| 2 to 4 | 111445.974(82017.905 to 144577.609) | 119.418(87.885 to 154.92) | 9055.998(5627.521 to 13522.149) | 9.135(5.677 to 13.641) | 8389.275(4921.01 to 12429.428) | 8.569(5.026 to 12.696) | 7344.293(4290.907 to 11134.737) | 7.604(4.443 to 11.529) | -8.577  (-8.885 to -8.267) | -0.168(-0.291 to -0.014) |
| 5 to 9 | 44735.344(25893.83 to 64411.625) | 29.917(17.317 to 43.076) | 9761.176(5313.93 to 16430.688) | 5.631(3.065 to 9.478) | 8221.828(4262.966 to 13643.298) | 4.781(2.479 to 7.934) | 7043.996(3683.13 to 11361.644) | 4.136(2.162 to 6.67) | -5.444  (-5.817 to -5.068) | -0.266(-0.363 to -0.154) |
| 10 to 14 | 20847.312(12852.102 to 31900.647) | 16.44(10.135 to 25.157) | 6983.702(4108.297 to 12005.811) | 3.917(2.305 to 6.735) | 6490.72(3836.084 to 10949.9) | 3.64(2.151 to 6.141) | 5935.153(3371.967 to 10056.506) | 3.332(1.893 to 5.647) | -4.859  (-5.125 to -4.592) | -0.149 (-0.234 to -0.051) |
| High-income | | | | | | | | | | |
| 0 to 14 | 1495.411(1405.479 to 1590.15) | 0.798(0.75 to 0.848) | 526.256(497.837 to 560.424) | 0.294(0.278 to 0.313) | 458.523(418.747 to 500.939) | 0.258(0.236 to 0.282) | 427.101(373.038 to 484.923) | 0.242(0.211 to 0.275) | -2.785  (-3.044 to -2.526) | -0.177(-0.263 to -0.094) |
| Neonatal | 130.733(122.761 to 140.765) | 13.708(12.873 to 14.76) | 85.992(80.883 to 92.12) | 10.555(9.928 to 11.307) | 78.164(70.568 to 85.848) | 9.766(8.817 to 10.726) | 73.182(62.656 to 83.71) | 9.176(7.856 to 10.496) | -0.341  (-0.585 to -0.096) | -0.131(-0.234 to -0.025) |
| 1 to 5 months | 694.436(639.093 to 753.221) | 13.206(12.154 to 14.324) | 188.616(174.571 to 204.305) | 4.159(3.849 to 4.505) | 168.325(152.742 to 186.77) | 3.786(3.435 to 4.2) | 158.508(136.339 to 182.175) | 3.588(3.086 to 4.124) | -3.928  (-4.172 to -3.682) | -0.137(-0.244 to -0.031) |
| 6 to 11 months | 259.679(232.647 to 287.32) | 4.222(3.783 to 4.672) | 72.659(64.793 to 81.371) | 1.348(1.202 to 1.51) | 56.781(48.558 to 66.081) | 1.079(0.923 to 1.255) | 51.298(42.728 to 60.945) | 0.986(0.822 to 1.172) | -3.177  (-3.507 to -2.845) | -0.268 (-0.36 to -0.169) |
| 12 to 23 months | 303.429(276.67 to 332.496) | 2.458(2.241 to 2.694) | 108.247(98.561 to 118.847) | 0.981(0.893 to 1.077) | 92.469(82.712 to 102.828) | 0.859(0.768 to 0.955) | 84.203(72.825 to 96.869) | 0.799(0.691 to 0.92) | -2.381  (-2.673 to -2.089) | -0.185(-0.269 to -0.098) |
| 2 to 4 | 50.841(46.598 to 55.138) | 0.138(0.126 to 0.15) | 28.081(26.124 to 30.069) | 0.081(0.075 to 0.087) | 23.636(21.244 to 25.841) | 0.069(0.062 to 0.076) | 19.975(17.767 to 22.25) | 0.06(0.053 to 0.067) | -1.069  (-1.435 to -0.702) | -0.259 (-0.33 to -0.191) |
| 5 to 9 | 36.134(28.559 to 45.867) | 0.058(0.046 to 0.073) | 25.596(23.518 to 27.991) | 0.042(0.039 to 0.046) | 22.73(20.761 to 24.816) | 0.038(0.035 to 0.041) | 23.546(21.261 to 25.971) | 0.039(0.036 to 0.043) | -0.183  (-0.607 to 0.242) | -0.071(-0.117 to -0.019) |
| 10 to 14 | 20.16(15.991 to 24.808) | 0.032(0.025 to 0.039) | 17.065(15.792 to 18.597) | 0.027(0.025 to 0.03) | 16.418(14.998 to 17.977) | 0.026(0.024 to 0.029) | 16.39(14.902 to 18.065) | 0.026(0.024 to 0.029) | 0.344  (-0.105 to 0.795) | -0.043(-0.078 to -0.005) |
| Central Europe, Eastern Europe, and Central Asia | | | | | | | | | | |
| 0 to 14 | 16048.808(14640.475 to 17683.295) | 15.149(13.82 to 16.692) | 2261.238(1708.928 to 2920.481) | 2.806(2.121 to 3.624) | 2095.225(1588.328 to 2757.971) | 2.592(1.965 to 3.412) | 2049.79(1545.178 to 2682.207) | 2.536(1.912 to 3.319) | -6.236  (-6.533 to -5.938) | -0.096(-0.171 to 0.011) |
| Neonatal | 1091.198(989.077 to 1211.703) | 215.691(195.506 to 239.511) | 188.55(142.855 to 253.595) | 48.276(36.576 to 64.93) | 176.629(132.635 to 239.876) | 46.332(34.792 to 62.922) | 171.863(127.025 to 236.995) | 46.258(34.189 to 63.788) | -6.239  (-6.692 to -5.784) | -0.042(-0.131 to 0.078) |
| 1 to 5 months | 4326.734(3838.112 to 4860.66) | 153.515(136.179 to 172.459) | 604.143 (438.513 to 823.936) | 27.83(20.2 to 37.955) | 574.292(414.027 to 796.035) | 27.106(19.541 to 37.572) | 556.488(397.752 to 776.502) | 26.937(19.253 to 37.587) | -7.834  (-8.441 to -7.223) | -0.032(-0.131 to 0.101) |
| 6 to 11 months | 6780.327(6084.79 to 7574.012) | 201.257(180.612 to 224.816) | 906.898(664.81 to 1193.573) | 35.189(25.796 to 46.313) | 828.389(585.211 to 1108.445) | 32.945(23.274 to 44.083) | 809.568(569.63 to 1076.989) | 32.992(23.214 to 43.889) | -6.902  (-7.406 to -6.394) | -0.062(-0.157 to 0.065) |
| 12 to 23 months | 2698.918(2383.508 to 3071.955) | 38.477(33.981 to 43.796) | 345.181(254.952 to 465.397) | 6.549(4.837 to 8.83) | 320.403(229.945 to 449.962) | 6.247(4.483 to 8.773) | 316.754(226.956 to 443.038) | 6.329(4.534 to 8.852) | -8.342  (-9.054 to -7.625) | -0.034 (-0.143 to 0.103) |
| 2 to 4 | 936.579(785.2 to 1101.322) | 4.221(3.538 to 4.963) | 156.828(109.261 to 225.205) | 0.944(0.658 to 1.356) | 138.482(93.248 to 206.691) | 0.853(0.574 to 1.273) | 137.675(91.118 to 210.481) | 0.871(0.577 to 1.332) | -6.901  (-7.487 to -6.312) | -0.077 (-0.196 to 0.082) |
| 5 to 9 | 152.204(130.712 to 172.573) | 0.425(0.365 to 0.482) | 41.156(26.883 to 59.756) | 0.147(0.096 to 0.213) | 39.239(25.033 to 58.338) | 0.139(0.089 to 0.207) | 39.381(24.567 to 60.302) | 0.14(0.087 to 0.214) | -5.293  (-5.93 to -4.651) | -0.046 (-0.137 to 0.079) |
| 10 to 14 | 62.849(54.844 to 70.107) | 0.184(0.16 to 0.205) | 18.482(13.233 to 24.753) | 0.072(0.052 to 0.097) | 17.79(12.529 to 24.349) | 0.068(0.048 to 0.093) | 18.059(12.605 to 24.935) | 0.067(0.047 to 0.092) | -4.521  (-4.985 to -4.055) | -0.076(-0.13 to -0.003) |
| Southeast Asia, East Asia, and Oceania | | | | | | | | | | |
| 0 to 14 | 257241.981(178710.822 to 322570.717) | 51.115(35.511 to 64.096) | 20523.036(15670.645 to 26355.241) | 4.619(3.527 to 5.931) | 18928.779(14478.759 to 24413.877) | 4.244(3.246 to 5.473) | 17810.68(13561.533 to 22762.359) | 4.002(3.047 to 5.114) | -7.683  (-7.827 to -7.538) | -0.134 (-0.214 to -0.04) |
| Neonatal | 24777.542(12586.765 to 36759.828) | 899.16(456.765 to 1333.989) | 1633.959(1198.863 to 2307.536) | 81.698(59.943 to 115.376) | 1493.576(1112.611 to 2079.906) | 80.727(60.136 to 112.418) | 1435.665(1060.168 to 1980.048) | 81.595(60.254 to 112.535) | -8.103  (-8.248 to -7.957) | -0.001(-0.08 to 0.088) |
| 1 to 5 months | 77857.321(54029.375 to 98214.061) | 516.564(358.472 to 651.626) | 6620.045(4832.052 to 8702.675) | 58.885(42.981 to 77.41) | 6191.855(4597.807 to 8198.114) | 59.374(44.089 to 78.612) | 5867.969(4399.716 to 7778.86) | 59.614(44.698 to 79.028) | -7.999  (-8.261 to -7.737) | 0.012(-0.089 to 0.121) |
| 6 to 11 months | 58780.128(42864.788 to 73326.687) | 336.136 (245.124 to 419.321) | 4776.81(3476.06 to 6489.167) | 35.068(25.519 to 47.638) | 4415.786(3204.001 to 5957.655) | 34.782(25.237 to 46.926) | 4086.968(2898.71 to 5558.325) | 34.56(24.512 to 47.002) | -7.649  (-7.762 to -7.535) | -0.014 (-0.134 to 0.13) |
| 12 to 23 months | 45849.16(31747.14 to 58153.429) | 131.043(90.737 to 166.21) | 3612.949(2653.137 to 4902.224) | 12.448(9.141 to 16.89) | 3303.446(2387.87 to 4502.495) | 12.14(8.775 to 16.546) | 3113.555(2250.643 to 4251.461) | 12.326(8.91 to 16.831) | -7.763  (-7.942 to -7.584) | -0.01(-0.121 to 0.13) |
| 2 to 4 | 37631.406(23202.537 to 50256.277) | 35.928(22.153 to 47.982) | 2126.979(1544.206 to 2909.317) | 2.266(1.645 to 3.1) | 1873.535(1348.494 to 2528.265) | 2.009(1.446 to 2.71) | 1755.029(1247.422 to 2398.706) | 1.959(1.392 to 2.677) | -8.933  (-9.013 to -8.853) | -0.136 (-0.235 to -0.016) |
| 5 to 9 | 7927.724(3955.38 to 13757.81) | 4.755(2.372 to 8.252) | 1065.137(646.099 to 1902.274) | 0.713(0.433 to 1.274) | 1002.822 (602.525 to 1763.807) | 0.655(0.394 to 1.152) | 954.09(576.946 to 1685.539) | 0.602(0.364 to 1.064) | -5.708  (-5.893 to -5.523) | -0.155(-0.241 to -0.059) |
| 10 to 14 | 4418.7(2311.083 to 8155.375) | 2.736(1.431 to 5.05) | 687.158(424.814 to 1203.932) | 0.473(0.293 to 0.829) | 647.76(393.682 to 1147.138) | 0.439(0.267 to 0.778) | 597.402(362.896 to 1060.456) | 0.403(0.245 to 0.714) | -5.164  (-5.574 to -4.752) | -0.149 (-0.241 to -0.047) |
| 0 to 14 | 94972.317(87666.942 to 102666.872) | 65.834(60.77 to 71.168) | 10182.003(8091.022 to 12723.912) | 7.045(5.599 to 8.804) | 9225.278(7022.359 to 11708.52) | 6.41(4.879 to 8.135) | 8760.027(6524.792 to 11263.551) | 6.114(4.554 to 7.861) | -7.401  (-7.77 to -7.032) | -0.132(-0.224 to -0.046) |
| Latin America and Caribbean | | | | | | | | | | |
| Neonatal | 6508.256(5876.499 to 7283.157) | 827.486 (747.162 to 926.01) | 641.43(439.86 to 879.008) | 86.84(59.55 to 119.005) | 597.611(401.479 to 834.614) | 82.552(55.459 to 115.291) | 578.788(383.659 to 819.208) | 81.339(53.917 to 115.125) | -7.151  (-7.841 to -6.455) | -0.063(-0.165 to 0.054) |
| 1 to 5 months | 38556.368(35238.3 to 41944.195) | 897.361 (820.136 to 976.209) | 3029.56(2230.081 to 3915.351) | 74.137(54.573 to 95.813) | 2738.907(1969.779 to 3644.072) | 68.365(49.167 to 90.959) | 2636.704(1887.23 to 3586.938) | 66.988(47.947 to 91.129) | -8.398  (-8.722 to -8.073) | -0.096(-0.201 to 0.018) |
| 6 to 11 months | 21816.152(19620.364 to 24208.061) | 437.861(393.79 to 485.868) | 2459.89(1790.135 to 3291.545) | 51.048(37.149 to 68.307) | 2352.285(1635.566 to 3172.265) | 49.784(34.615 to 67.138) | 2218.634(1544.118 to 3038.815) | 47.841(33.296 to 65.527) | -7.222  (-7.501 to -6.942) | -0.063(-0.183 to 0.065) |
| 12 to 23 months | 17680.032(16168.797 to 19363.83) | 178.185(162.954 to 195.154) | 2510.714(1949.205 to 3140.949) | 25.831(20.054 to 32.315) | 2208.679(1668.23 to 2878.77) | 23.134(17.473 to 30.152) | 2092.061(1529.761 to 2736.441) | 22.32(16.321 to 29.195) | -6.194  (-6.512 to -5.874) | -0.136(-0.237 to -0.035) |
| 2 to 4 | 7157.857(6247.655 to 8065.53) | 24.246(21.163 to 27.32) | 869.839 (638.328 to 1194.529) | 2.967(2.178 to 4.075) | 722.626(506.813 to 1036.985) | 2.489(1.746 to 3.572) | 663.26(454.773 to 959.847) | 2.314(1.587 to 3.349) | -7.126  (-7.496 to -6.755) | -0.22(-0.331 to -0.096) |
| 5 to 9 | 2304.879(2041.607 to 2586.001) | 4.749(4.207 to 5.329) | 435.976(348.257 to 556.801) | 0.905(0.723 to 1.155) | 389.233(297.34 to 505.27) | 0.807(0.617 to 1.048) | 362.572(276.469 to 471.094) | 0.753(0.574 to 0.978) | -5.176  (-5.724 to -4.625) | -0.168(-0.237 to -0.101) |
| 10 to 14 | 948.773(843.427 to 1065.249) | 2.053(1.825 to 2.305) | 234.595(194.649 to 286.689) | 0.492(0.409 to 0.602) | 215.937(175.741 to 268.596) | 0.453(0.369 to 0.563) | 208.008(166.974 to 263.121) | 0.435(0.349 to 0.551) | -4.638  (-5.207 to -4.066) | -0.116 (-0.173 to -0.058) |
| Sub-Saharan Africa | | | | | | | | | | |
| 0 to 14 | 598844.611(419893.07 to 750169.711) | 266.819 (187.086 to 334.243) | 306262.137(224212.497 to 419556.91) | 66.482(48.671 to 91.076) | 279199.644(198260.249 to 389673.76) | 59.591(42.316 to 83.17) | 264036.939(186466.383 to 372856.373) | 55.477(39.178 to 78.341) | -4.768  (-5.095 to -4.44) | -0.166(-0.219 to -0.113) |
| Neonatal | 38872.298(24120.222 to 51075.237) | 2353.924(1460.607 to 3092.876) | 16342.112(12266.579 to 22115.074) | 587.072(440.663 to 794.459) | 14729.519(10880.561 to 20306.229) | 526.569(388.972 to 725.932) | 14290.738(10429.957 to 19758.984) | 506.312 (369.527 to 700.048) | -4.5  (-4.841 to -4.159) | -0.138(-0.198 to -0.088) |
| 1 to 5 months | 163479.704(119598.419 to 196037.893) | 1850.299(1353.641 to 2218.8) | 81330.515(62828.154 to 105265.387) | 535.653(413.794 to 693.291) | 71877.541(53997.728 to 93406.738) | 470.709(353.618 to 611.698) | 69398.571(52100.837 to 90870.118) | 450.771(338.415 to 590.237) | -4.369  (-4.714 to -4.023) | -0.158(-0.209 to -0.11) |
| 6 to 11 months | 126191.324(90920.17 to 159831.328) | 1265.497(911.784 to 1602.852) | 74270.925(51774.12 to 107817.914) | 422.993(294.868 to 614.053) | 67631.749(45055.078 to 99676.577) | 382.527(254.833 to 563.774) | 63731.123(42991.686 to 94681.87) | 358.22(241.648 to 532.188) | -3.619  (-3.87 to -3.368) | -0.153 (-0.218 to -0.086) |
| 12 to 23 months | 126589.999(84560.995 to 163048.066) | 676.035(451.585 to 870.734) | 59649.44(41501.884 to 83913.32) | 172.913(120.307 to 243.25) | 53901.159(36339.853 to 78350.438) | 154.848(104.397 to 225.086) | 50211.383(33182.85 to 73735.462) | 143.202(94.637 to 210.292) | -4.821  (-5.074 to -4.567) | -0.172(-0.242 to -0.096) |
| 2 to 4 | 123432.039(77616.722 to 167724.338) | 244.399(153.683 to 332.099) | 55537.831(34956.357 to 83720.546) | 55.842(35.148 to 84.179) | 52466.676(31421.022 to 80034.665) | 52.119(31.213 to 79.505) | 48334.992(28398.75 to 75658.705) | 47.491(27.903 to 74.337) | -5.242  (-5.524 to -4.959) | -0.15(-0.222 to -0.078) |
| 5 to 9 | 14055.699(7382.988 to 23006.324) | 19.256(10.114 to 31.518) | 13050.255(7219.526 to 21863.525) | 8.476(4.689 to 14.2) | 12607.947(6805.119 to 21533.314) | 8.051(4.345 to 13.75) | 12171.819(6583.071 to 20704.083) | 7.651(4.138 to 13.014) | -2.552  (-2.739 to -2.364) | -0.097(-0.169 to -0.043) |
| 10 to 14 | 6223.548(3361.307 to 10335.717) | 10.078(5.443 to 16.736) | 6081.058(3429.453 to 10338.059) | 4.431(2.499 to 7.534) | 5985.054(3448.218 to 9931.306) | 4.254(2.451 to 7.059) | 5898.314(3356.393 to 9838.493) | 4.096(2.331 to 6.832) | -2.57  (-2.762 to -2.377) | -0.076(-0.128 to -0.017) |

Values in parentheses are 95% uncertainty intervals. Count data are presented to three significant figures. GBD=Global Burden of Diseases, Injuries, and Risk Factors Study. SDI=Socio-demographic Index.
